# Identification of a Comprehensive Spectrum of Genetic Factors for Hereditary Breast Cancer in a Chinese Population by Next-Generation Sequencing

**DOI:** 10.1371/journal.pone.0125571

**Published:** 2015-04-30

**Authors:** Xiaochen Yang, Jiong Wu, Jingsong Lu, Guangyu Liu, Genhong Di, Canming Chen, Yifeng Hou, Menghong Sun, Wentao Yang, Xiaojing Xu, Ying Zhao, Xin Hu, Daqiang Li, Zhigang Cao, Xiaoyan Zhou, Xiaoyan Huang, Zhebin Liu, Huan Chen, Yanzi Gu, Yayun Chi, Xia Yan, Qixia Han, Zhenzhou Shen, Zhimin Shao, Zhen Hu

**Affiliations:** 1 Department of Breast Surgery, Shanghai Cancer Center, Fudan University, Shanghai, China; 2 Department of Oncology, Shanghai Medical College, Fudan University, Shanghai, China; 3 Tissue Bank, Department of Pathology, Shanghai Cancer Center, Fudan University, Shanghai, China; 4 Department of Pathology, Shanghai Cancer Center, Fudan University, Shanghai, China; 5 Laboratory of molecular biology, National Engineering Center for Biochip at Shanghai, Shanghai, China; 6 Key Laboratory of Breast Cancer in Shanghai, Shanghai Cancer Center, Fudan University, Shanghai, China; 7 Institute of Pathology, Fudan University, Shanghai, China; CNR, ITALY

## Abstract

The genetic etiology of hereditary breast cancer has not been fully elucidated. Although germline mutations of high-penetrance genes such as BRCA1/2 are implicated in development of hereditary breast cancers, at least half of all breast cancer families are not linked to these genes. To identify a comprehensive spectrum of genetic factors for hereditary breast cancer in a Chinese population, we performed an analysis of germline mutations in 2,165 coding exons of 152 genes associated with hereditary cancer using next-generation sequencing (NGS) in 99 breast cancer patients from families of cancer patients regardless of cancer types. Forty-two deleterious germline mutations were identified in 21 genes of 34 patients, including 18 (18.2%) BRCA1 or BRCA2 mutations, 3 (3%) TP53 mutations, 5 (5.1%) DNA mismatch repair gene mutations, 1 (1%) CDH1 mutation, 6 (6.1%) Fanconi anemia pathway gene mutations, and 9 (9.1%) mutations in other genes. Of seven patients who carried mutations in more than one gene, 4 were BRCA1/2 mutation carriers, and their average onset age was much younger than patients with only BRCA1/2 mutations. Almost all identified high-penetrance gene mutations in those families fulfill the typical phenotypes of hereditary cancer syndromes listed in the National Comprehensive Cancer Network (NCCN) guidelines, except two TP53 and three mismatch repair gene mutations. Furthermore, functional studies of MSH3 germline mutations confirmed the association between MSH3 mutation and tumorigenesis, and segregation analysis suggested antagonism between BRCA1 and MSH3. We also identified a lot of low-penetrance gene mutations. Although the clinical significance of those newly identified low-penetrance gene mutations has not been fully appreciated yet, these new findings do provide valuable epidemiological information for the future studies. Together, these findings highlight the importance of genetic testing based on NCCN guidelines and a multi-gene analysis using NGS may be a supplement to traditional genetic counseling.

## Introduction

Germline mutations in *BRCA1* (MIM# 113705) and *BRCA2* (MIM# 600185) are the most common cause of hereditary breast cancer, and early diagnosis and preventive interventions have been confirmed to improve the survival rate of mutation carriers [1]. However, *BRCA1* and *BRCA2* germline mutations are present in only half of all families with a strong family history of breast cancer. Other high-penetrance genes that also play an important role in the genetic etiology of breast cancer include *TP53*, *PTEN* and *CDH1* [2–4]. Additionally, germline mutations in many moderate-to-low-penetrance genes can also lead to hereditary breast cancer [5].

In clinical practice, the families of breast cancer probands present with multiple types of malignant tumors that are not limited to breast and ovarian cancers. Moreover, the correlation between breast cancer and several other hereditary cancer syndromes remains unclear. Breast cancer may not represent the primary phenotype of these hereditary cancer syndromes; however, it occurs frequently [6]. In traditional cancer genetic counseling, prior to genetic testing, families are divided into different hereditary cancer syndromes based on the characteristics of the probands and their family history. Subsequently, the related genes are tested to determine the etiology of the disease. This approach is useful for identifying high-risk populations and susceptibility genes and for improving detection efficiency; however, some individuals may not be successfully identified using this method [7, 8]. Currently, a mature genetic counseling system in China is not available, and there is a lack of relevant systematic studies.

In recent years, the rapid development of next-generation sequencing (NGS) technologies has enabled the simultaneous sequencing of multiple genes, and the cost is comparable to that of single-gene sequencing using traditional sequencing techniques. Studies have suggested that such cost-effective approach could be used to the genetic testing and personalized risk assessment for individuals at high risk of breast cancer [9]. Our study utilized two important approaches. First, we randomly selected family members of breast cancer probands and did not limit the cancer types in the family history. Second, we selected 152 genes associated with hereditary cancer and performed comprehensive testing using NGS. Through these efforts, we aimed to obtain comprehensive results regarding the genes for hereditary breast cancer susceptibility in the Chinese population and to lay a foundation for the development of cancer genetic counseling in China. The value of NGS applied in genetic counseling also needed to be evaluated.

## Materials and Methods

### Ethics statement

This study was approved by the Scientific and Ethical Committee of the Shanghai Cancer Center, Fudan University. Written informed consent was obtained from all participants.

### Patient/Sample Selection

Breast cancer patients were consecutively enrolled after diagnosis at the Shanghai Cancer Center of Fudan University from June 2011 to July 2012. To be eligible for the study, the breast cancer patients were required to meet one of the following inclusion criteria:

1. Age younger than or equal to 35 years with at least one other blood relative suffering from any type of cancer
2. Age older than 35 and younger than or equal to 50 years with ≥2 blood relatives in the same lineage suffering from any type of cancer
3. Age older than 50 years with ≥3 blood relatives in the same lineage suffering from any type of cancer.

Pedigrees and medical records were collected via questionnaires at study entry. Pathologies and other medical reports were reviewed for the probands and their blood relatives when available. For each patient, blood and formalin-fixed paraffin-embedded (FFPE) specimens were collected from each patient, and samples from relatives of the probands who carried candidate mutations were obtained when available. Genomic DNA was extracted using a Chemagic STAR DNA Blood 4k Kit (PerkinElmer, Waltham, Massachusetts, USA) or a QIAamp DNA FFPE Tissue Kit (Qiagen, Hilden, Germany), according to the manufacturer’s recommended protocol.

### Gene Selection

In total, 152 genes were tested, selected as cancer susceptibility genes based on a PubMed keyword search of ("Neoplasms” [Mesh] AND “germline mutation”) in 8334 studies published up to June 2012. Among these genes, 115 are known to be associated with hereditary cancer syndromes. A summary of the genes is provided in S1 Table.

### Targeted Capture and Massively Parallel Sequencing

A sample of 3 μg of genomic DNA was fragmented to 150–200 bp using sonication (Covaris Inc., Woburn, MA), and adapters were subsequently ligated to both ends of the resulting fragments. After purification and amplification, target-region capture was performed using the pre-designed Human SureSelect XT Custom Kit (700 Kb-34 Mb, Agilent Technologies) according to the recommended protocols. Captured libraries were analyzed using an Agilent 2100 Bioanalyzer to estimate the magnitude of enrichment and were sequenced with 90-bp paired end reads on a HiSeq2000 platform subsequently (Illumina, San Diego, CA, USA). We independently performed high-throughput sequencing of each captured library to ensure that each sample met the desired average fold coverage. Coverage was calculated by counting the number of sequenced bases that mapped to the target regions. Bases mapping to regions within a 200-bp range of a target were considered “near target”.

### Bioinformatic Analysis of DNA Variants

Raw image files were processed using Illumina base-calling software 1.7 with default parameters, and the sequences for each individual were generated as 90-bp paired-end reads. After filtering, high-quality reads were aligned to the human genome (GRCH37, UCSC hg19) using the Burrows-Wheeler Aligner program [10]. SAMtools [11] and SOAPsnp [12] were used for the identification of small insertions or deletions (InDels) and single nucleotide polymorphisms (SNPs), respectively. SNPs were called using SOAPsnp with options:-r 0.00005-e 0.0001-t-u-L 90-Q L. Next, filters of quality score (≥20), neighbor distance (≥5) and depth (≥4) were applied to the SNPs calling results. Finally, ANNOVAR [13] was used for the annotation of InDels and SNPs.

### Filtering of Variants

Variants were selected for follow-up when all of the following criteria applied:

1. Variants were located in the exonic region or were within -2 bp away from an exon/intron boundary (splicing).
2. Variants were InDels, nonsynonymous (missense or nonsense) or splicing variants.
3. Variants did not occur within the 1000 Genomes (1000G) data with a minor allele frequency (MAF) greater than 0.01[14].
4. Variants were consistent with the known pattern of inheritance of the respective gene.

If prioritization resulted in a single strong candidate allele (a nonsense or canonical splice site variant) in a recessively acting gene, a manual literature search was performed to confirm the pathogenicity of the heterozygous mutation.

### Determination of Pathogenicity of Variants

Frameshift InDels, non-frameshift InDels, nonsense mutations, and splicing mutations were considered to be pathogenic. For missense mutations, we developed a classification system based on *in silico* evidence, which contained two different features: (1) missense prediction software (acting at the amino acid level) and (2) evolutionary conservation (acting at the nucleotide level).

1. In the case of missense prediction programs, SIFT [15] and PolyPhen-2 [16] were used. A variant was considered to be “damaging” when either prediction program predicted it as “damaging.”
2. For classification based on evolutionary conservation, LJB_PhyloP [17] and LJB_LRT [18] were used. A variant was considered to be “damaging” when either prediction program predicted it as “conserved.”

Variants considered to be “damaging” by both classifications (prediction tools and conservation) were predicted as “damaging” and were manually referenced to gene-specific mutation databases or published studies. Those with clear pathogenic impact, as reported by previous studies, were selected for further analysis.

### Variant Validation and Segregation Analysis

All candidate variants were manually referenced to gene-specific mutation databases or published studies and were validated using conventional Sanger sequencing (ABI PRISM 3730XL Genetic Analyzer). Where available, DNAs from additional family members were sequenced to enable segregation analysis. The sequencing results were evaluated using Chromas software, version 2.4.1 (Technelysium Pty Ltd., South Brisbane QLD, Australia).

### Functional Studies of MSH3 Germline Mutation

For patients with mutations in the DNA mismatch repair (*MMR*) gene *MSH3*, the expression of Msh3 protein was evaluated by immunohistochemistry (IHC) using an anti-Msh3 rabbit monoclonal antibody (clone EPPR4334(2); Epitomics, USA) at a dilution of 1:500. The expression of Msh3 was scored by two specialized pathologists (W.T.Y. and X.Y.Z.) as follows: 0, no staining; 1+, weak staining; 2+, intermediate staining; and 3+, strong staining. Microsatellite instability (MSI) was determined using fluorescent multiplex PCR of paired normal and tumor DNA samples by employing the MSI analysis system on an ABI PRISM 3500 Avant Genetic Analyzer (Applied Biosystems, Carlsbad, California, USA). Fluorescent multiplex PCR was performed using the RT-PCR Quick Master Mix Kit (Toyobo, Osaka, Japan). Five consensus National Cancer Institute (NCI) microsatellite markers (BAT25, BAT26, D2S123, D5S346, and D17S250), two additional dinucleotide repeat markers (D18S64 and D18S69) and seven elevated microsatellite alterations at selected tetra-nucleotide repeat (EMAST) markers (MYCL1, D20S82, D20S85, L17835, D8S321, D9S242 and D19S394) were used. The results were analyzed by two specialized pathologists (W.T.Y. and X.Y.Z.). The criteria for the determination of MSI were those described by Haugen et al. [19].

### Statistical Analysis

The average age of onset of patients carrying a single *BRCA1/2* mutation and those carrying other gene mutations in addition to a *BRCA1/2* mutation were compared using a *t*-test. The analysis was performed using SPSS software, version 19.0 (IBM institute). All P values were two-sided, and any P value <0.05 was considered significant.

### Accession Codes

The GenBank reference sequences used for variant annotation are summarized in S2 Table. Nucleotide numbering reflects cDNA numbering with +1 corresponding to the A of the ATG translation initiation codon in the reference sequence. The initiation codon is codon 1.

## Results

### Patient Characteristics

**Fig 1:**
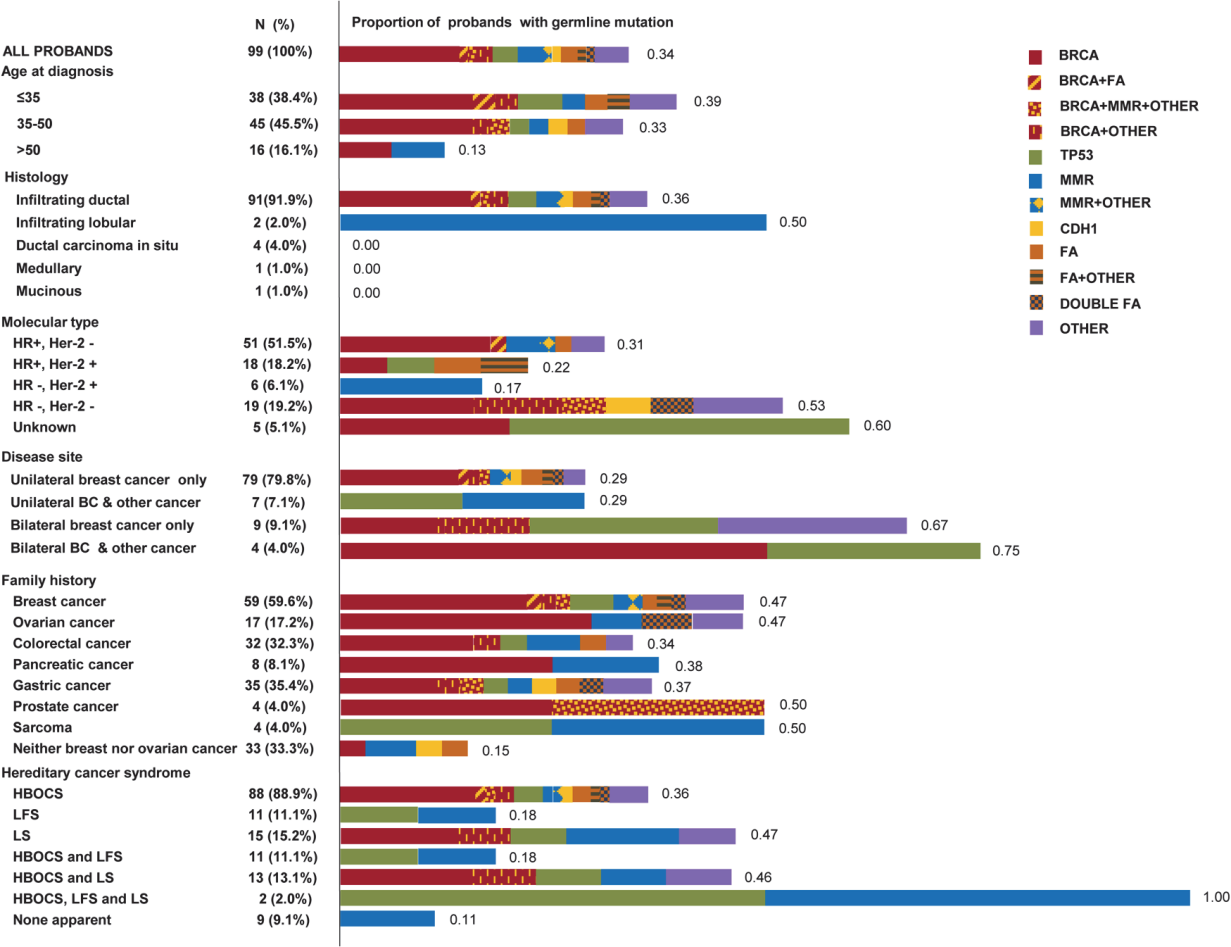
Characteristics of 99 probands and their families, and proportions of probands with germline deleterious mutations. This figure consisted of two aspects. The left column presented the overview of characteristics of the 99 probands and their families. It provided the numbers and proportions of each subgroup. The right color lumps reflected the proportions of probands with different gene mutations in each subgroup. The numbers on the right side of the color lumps represented the proportions. Proportions of probands with one gene mutation are shown in pure color squares, and proportions of probands with mutations in two or more genes are shown in two-color squares. Abbreviations and definitions are as follows: HR, hormone receptor; Her-2, human epidermal growth factor receptor 2; BC, breast cancer; HBOCS, hereditary breast and ovarian cancer syndrome; LFS, Li-Fraumeni syndrome; LS, Lynch syndrome; FA, Fanconi anemia genes, including *RAD50*, *PALB2*, *FANCD2*, *FANCI*, *SLX4*, and *RAD51C*. *MMR*, mismatch repair genes, including *MLH1*, *MLH3*, and *MSH3*. OTHER, other genes, including *RGSL1*, *CDKN2A*, *SPINK1*, *TNFRSF13B*, *FGFR3*, *WRN*, *MUTYH*, and *CYP17A1*.

Of the 3,102 breast cancer patients who underwent surgery in our hospital, 134 met the inclusion criteria. Thirty-five patients refused enrollment, and 99 patients entered the study. These patients are all independent with no blood relationship. They are recruited from families with at least 2 relatives suffering from certain type of cancer to ensure that germline mutations, rather than somatic mutations or environmental exposures, are the determinations of cancer. The characteristics of the 99 probands and their families are presented in Fig 1. Infiltrating ductal carcinoma was the most prominent histological type and accounted for approximately 92% of all types. Thirteen patients exhibited bilateral disease, and 11 patients in addition to breast cancer also suffered from another malignant tumor. Breast cancer was the most common malignancy within the family history (59.6%). According to the National Comprehensive Cancer Network (NCCN) guidelines (version 4.2013), 88, 11 and 15 families fulfilled the criteria for hereditary breast and ovarian cancer syndrome (HBOCS), Li-Fraumeni syndrome (LFS, according to classic LFS criteria or Chompret criteria), and Lynch syndrome (LS, according to the revised Bethesda criteria), respectively.

### Mutation Detection and Characterization

**Fig 2:**
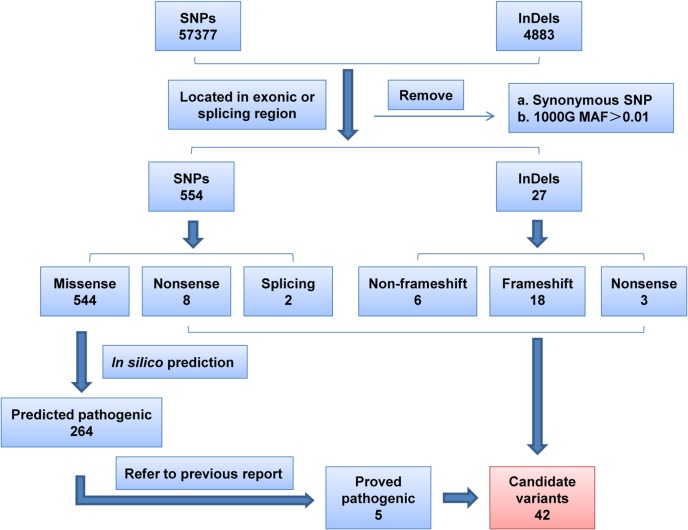
Filtering of variants. Detailed procedure was described in *Materials and Methods*. The URLs for the locus-specific databases used were as follows: *BRCA1/2*, http://research.nhgri.nih.gov/bic/; *TP53*, http://www-p53.iarc.fr/; DNA *MMR* genes, http://www.insight-group.org/.

**Fig 3:**
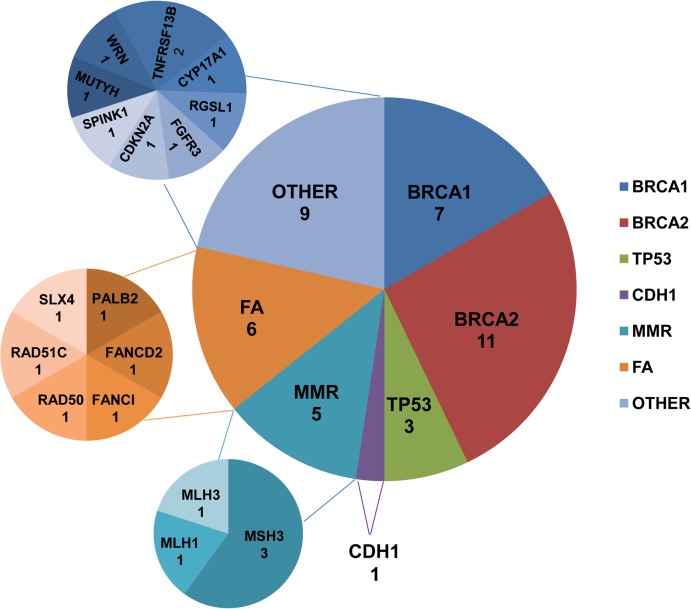
Number of germline loss-of-function mutations identified in different genes. Abbreviations: MMR, mismatch repair; FA, Fanconi anemia.

A total of 99 breast cancer patients were screened for germline mutations in 2,165 coding exons of 152 genes. The average sequencing depths of the targeted regions were 93 to 123. Over 98% coverage of the targeted regions was achieved for each proband. The coverage of the targeted exons for the >20× reads ranged from 87.60% to 92.60% and its standard deviations was 0.93%. Thus, the coverage should have been adequate to reliably detect DNA variants within the majority of the targeted regions. The NGS results, including the number of reads, sample coverage, and sequencing depth, are summarized in S3 Table. Overall, 4883 InDels and 57377 SNPs were obtained. Systematic filtering of variants was accomplished as described in *Materials and Methods* and is summarized in Fig 2. A total of 42 putative deleterious germline mutations in 21 genes (Fig 3) were identified in 34 breast cancer patients, including 22 frameshift InDels, 5 non-frameshift InDels, 8 nonsense mutations, 5 missense mutations and 2 splicing mutations. Features of the mutations, family characteristics, segregation analysis and age of diagnosis for each of the patients carrying a mutation are provided in Table 1.

**Table 1:** All putative deleterious germline mutations and proband characteristics.

ID	Age dx (yrs)	HCS met	Gene	DNA change	Protein change	Chr	Mutant site start	Mutant site end	Segregation^a^	1000G MAF^b^	Missense prediction SIFT^c^	Missense prediction PolyPhen-2^c^	Evolutionary conservation PhyloP^c^	Evolutionary conservation LRT^c^	Reference
56	35	HBOCS	*BRCA1*	c.754delC	p.R252Vfs*46	17	41246794	41246794	2/2	0	—	—	—	—	Novel
6	32	HBOCS	*BRCA1*	c.1214C>G	p.S405*	17	41246334	41246334	2/3	0	—	—	—	—	Novel
22	37	HBOCS	*BRCA1*	c.2110_2111delAA	p.N704Cfs*7	17	41245438	41245439	5/6	0	—	—	—	—	BIC
5	40	HBOCS	*BRCA1*	c.4228delG	p.E1410Kfs*5	17	41234551	41234551	3/4	0	—	—	—	—	Novel
29	39	HBOCS	*BRCA1*	c.5503C>T	p.R1835*	17	41197784	41197784	3/3	0	—	—	—	—	BIC
26	40	HBOCS	*BRCA1*	c.5468-1_5474del8	p.A1823Gfs*9	17	41197813	41197820	3/3	0	—	—	—	—	[33]
52	34	HBOCS	*BRCA1*	c.5468-1_5474del8	p.A1823Gfs*9	17	41197813	41197820	6/6	0	—	—	—	—	[33]
3	46	HBOCS	*BRCA2*	c.2442delC	p.M815Wfs*10	13	32910934	32910934	2/3	0	—	—	—	—	[33, 54]
4	42	HBOCS, LS	*BRCA2*	c.2442delC	p.M815Wfs*10	13	32910934	32910934	3/3	0	—	—	—	—	[33, 54]
68	38	HBOCS	*BRCA2*	c.2808_2811delACAA	p.A938Pfs*21	13	32911300	32911303	U/A	0	—	—	—	—	BIC
2	37	HBOCS	*BRCA2*	c.5682C>G	p.Y1894*	13	32914174	32914174	2/2	0	—	—	—	—	BIC
7	30	HBOCS	*BRCA2*	c.5699C>G	p.S1900*	13	32914191	32914191	1/2	0	D	B	C	NC	Novel
54	70	HBOCS	*BRCA2*	c.7007G>A	p.R2336H	13	32921033	32921033	U/A	0	D	B	C	NC	BIC
24	26	HBOCS, LS	*BRCA2*	c.7142delC	p.P2381Hfs*13	13	32929132	32929132	5/6	0	—	—	—	—	Novel
45	43	HBOCS	*BRCA2*	c.7409dupT	p.T2471Hfs*4	13	32929399	32929399	U/A	0	—	—	—	—	[31]
42	34	HBOCS, LS	*BRCA2*	c.8485C>T	p.Q2829*	13	32944692	32944692	2/2	0	—	—	—	—	BIC
13	32	HBOCS	*BRCA2*	c.8517C>A	p.Y2839*	13	32945122	32945122	U/A	0	D	B	C	C	Novel
71	28	HBOCS	*BRCA2*	c.8956_8957insAA	p.I2986Kfs*3	13	32953889	32953889	U/A	0	—	—	—	—	Novel
65	30	HBOCS,LFS, LS	*TP53*	c.320dupA	p.Y107*	17	7579367	7579367	2/2	0	—	—	—	—	Novel
83	20	HBOCS	*TP53*	c.523C>G	p.R175G	17	7578407	7578407	2/2	0	D	PD	C	C	p53 mutation database
38	37	HBOCS	*TP53*	c.839G>C	p.R280T	17	7577099	7577099	U/A	0	D	PD	C	C	p53 mutation database
46	52	HBOCS, LS	*MLH1*	c.194G>A	p.G65D	3	37038187	37038187	U/A	0	D	PD	C	C	[55]
67	46	HBOCS	*MLH3*	c.1189_1191delTAT	p.I397del	14	75515169	75515171	U/A	0	—	—	—	—	Novel
22	37	HBOCS	*MSH3*	c.162_179del18	p.A57_A62del	5	79950708	79950725	5/6	0	—	—	—	—	Novel
49	32	HBOCS,LFS, LS	*MSH3*	c.199_207del9	p.P67_P69del	5	79950745	79950753	5/5	0	—	—	—	—	rs3045983
75	51	HBOCS	*MSH3*	c.2305delG	p.V769*	5	80071564	80071564	2/3	0	—	—	—	—	Novel
94	47	HBOCS	*CDH1*	c.1296C>G	p.N432K	16	68847374	68847374	U/A	0	D	PD	NC	C	[47]
86	52	HBOCS	*RAD51C*	c.343dupG	p.V115Gfs*24	17	56772489	56772489	2/2	0	**—**	**—**	**—**	**—**	Novel
51	45	HBOCS	*PALB2*	c.1050_1051delinsTCT	p.Q350Hfs*11	16	23646815	23646815	3/4	0	**—**	**—**	**—**	**—**	[56]
77	34	HBOCS	*RAD50*	c.1291_1295delGAGAT	p.E431Kfs*3	5	131925368	131925372	1/2	0	**—**	**—**	**—**	**—**	Novel
35	30	HBOCS	*FANCI*	c.2699_2704dupGGCAAT	p.Q901_F902insWQ	15	89843611	89843611	U/A	0	**—**	**—**	**—**	**—**	Novel
86	52	HBOCS	*SLX4*	c.3583_3585delATT	p.I1195del	16	3640054	3640056	1/2	0	**—**	**—**	**—**	**—**	rs199897550
7	30	HBOCS	*FANCD2*	c.4234_4239delAGTGAG	p.S1412_E1413del	3	10140452	10140457	1/2	0	**—**	**—**	**—**	**—**	Novel
77	34	HBOCS	*CDKN2A*	c.480G>A	p.W160*	9	21968748	21968748	1/2	0	**—**	**—**	**—**	**—**	Novel
64	27	HBOCS	*CYP17A1*	c.987delC	p.Y329*	10	104592420	104592420	U/A	0	**—**	**—**	**—**	**—**	Novel
88	45	HBOCS	*FGFR3*	c.2072delG	p.G691Afs*17	4	1808976	1808976	1/3	0	**—**	**—**	**—**	**—**	Novel
75	51	HBOCS	*RGSL1*	c.1357C>T	p.Q453*	1	182443603	182443603	2/3	0	**—**	**—**	**—**	**—**	Novel
22	37	HBOCS	*MUTYH*	c.850-2A>G	p.?	1	45797760	45797760	1/2	0.002	—	—	—	**—**	[57]
96	35	HBOCS	*SPINK1*	c.194+2T>C	p.?	5	147207583	147207583	2/2	0.001	—	—	—	**—**	[58]
60	47	HBOCS, LS	*TNFRSF13B*	c.704_705delCT	p.P235Rfs*169	17	16855857	16855857	2/2	0	—	—	—	**—**	Novel
29	39	HBOCS	*TNFRSF13B*	c.102delC	p.E36Kfs*48	17	16843038	16843039	3/3	0	—	—	—	**—**	Novel
24	26	HBOCS, LS	*WRN*	c.4245dupT	p.D1416*	8	31030564	31030564	5/6	0	—	—	—	**—**	Novel

Mutation nomenclature follows the recommended guidelines of the Human Genome Variation Society, with the nucleotide numbering based on the GenBank reference sequence indicated by its accession NCBI number. Details are listed in S2 Table.

Abbreviations: HCS, hereditary cancer syndrome; HBOCS, hereditary breast and ovarian cancer syndrome; LFS, Li-Fraumeni syndrome; LS, Lynch syndrome; U/A, unavailable; D, damaging; B, benign; C, conserved; NC, not conserved; and PD, probably damaging. BIC, breast cancer information core, URL: http://research.nhgri.nih.gov/bic/. p53 mutation database, URL: http://www-p53.iarc.fr, version R16.

^a^ Results of segregation studies marking positive individuals out of the total number available for validation.

^b^ All variants were queried against 1000 Genomes (1000G) data using the 1000 Genomes Browser (http://browser.1000genomes.org/index.html) which integrates SNP and InDel calls from 1,092 individuals (data released 2012 April). The minor allele frequency (MAF) is provided here.

^c^ *In sillico* prediction (missense prediction software and evolutionary conservation) were used for the determination of pathogenicity of missense mutations. Detailed criteria were described in *Materials and Methods*.

### Mutation Analyses of *BRCA1/2*, *TP53*, and *MMR* Genes

Eighteen (18.2%) patients were found to carry *BRCA1/2* germline mutations (7 in *BRCA1* and 11 in *BRCA2*) (Table 1). Two recurrent mutations, *BRCA1* c.5468-1_5474del8 and *BRCA2* c.2442delC, were each identified in two unrelated patients. Based on their family history, all patients with *BRCA1/2* mutations met the criteria for HBOCS, and these patients accounted for 20.5% (18/88) of the families who fulfilled the HBOCS criteria. The frequency of triple-negative breast cancer was 71.4% (5/7) in *BRCA1* mutation carriers and 9.1% (1/11) in *BRCA2* mutation carriers.

Three *TP53* germline mutations were identified. One was novel and another two were detected within the germline and somatic mutations in the p53 mutation database, respectively [20] (Table 1). One family with *TP53* mutation met the criteria for LFS and accounted for 9.1% of all families who fulfilled the LFS criteria. Two of the carriers were diagnosed with breast cancer under the age of 30, and they accounted for 10% (2/20) of all very young (≤30 years) breast cancer patients in our study. One carrier of a *TP53* mutation whose family history did not meet the LFS criteria was diagnosed at age 37 and reported a family history of malignant phyllodes tumor of the breast (PTB). Another *TP53* mutation carrier whose family met the criteria for LFS also reported a family history of PTB.

Deleterious mutations within the *MMR* genes *MLH1*, *MLH3*, and *MSH3* were detected in five distinct families. Three mutations were novel and two were previously reported (Table 1). Two families met the criteria for LS and accounted for 13.3% (2/15) of the families fulfilling the LS criteria.

In summary, all *BRCA1/2* mutations occurred in patients with a family history of HBOCS, but two *TP53* mutations occurred in patients without a family history of LFS, and three *MSH3* mutations occurred in patients without a family history of LS (Table 2).

**Table 2:** Analysis of *BRCA1/2*, *TP53*, and *MMR* gene mutation carriers fulfilling and not fulfilling the HBOCS, LFS, and LS criteria according to the NCCN guidelines.

	*BRCA1/2*	*TP53*	*MMR* gene
**No. of mutation carriers identified in this study using NGS**	18	3	5
**No. of mutation carriers fulfilling the HBOCS, LFS or LS criteria** ^*^	18	1	2
**No. of mutation carriers not fulfilling the HBOCS, LFS or LS criteria** ^*^	0	2	3

Abbreviations: HBOCS, hereditary breast and ovarian cancer syndrome; LFS, Li-Fraumeni syndrome; LS, Lynch syndrome; NCCN, national comprehensive cancer network; *MMR*, mismatch repair; and NGS, next-generation sequencing.

^*^ HBOCS, LFS and LS criteria used were according to the NCCN guidelines (version 4.2013).

### Mutation Analyses of Other Genes

One deleterious mutation was found in *CDH1* and this mutation has been previously reported (Table 1). The patient carrying the *CDH1* mutation had infiltrating ductal carcinoma of the breast, and her two blood relatives in the same lineage were diagnosed with gastric cancer; however, the histological types of the two gastric cancers were unknown.

Six Fanconi anemia (FA) pathway genes were found to carry loss-of-function mutations (Table 1). These genes were *RAD50*, *PALB2*, *FANCD2*, *FANCI*, *SLX4* and *RAD51C*. They accounted for 31.6% (6/19) of all of the non-*BRCA* mutation genes, which confirmed that multiple genes in the FA pathway are associated with breast cancer risk [21].

Nine deleterious mutations were identified in eight other genes (Table 1), *RGSL1*, *CDKN2A*, *SPINK1*, *TNFRSF13B*, *FGFR3*, *WRN*, *MUTYH* and *CYP17A1*. The last three genes exhibit an autosomal recessive mode of inheritance. Studies have reported that these gene mutations can also increase the risk of breast cancer or other cancers [22–29].

### Patients with Mutations in Two or More Genes

Seven patients were found to carry mutations in two or more genes (20.6%, 7/34). Among these patients, four were carriers of *BRCA1/2* mutations. Details about the patients carrying mutations in two or more genes are provided in Table 3 and Fig 1. In comparison to patients with single mutations in *BRCA1/2*, the average age of onset of patients carrying mutations in other genes in addition to *BRCA1/2* was 7 years earlier, regardless of whether the comparison was made among the probands alone (40.6 years vs. 33.3 years, P = 0.22) or together with their blood relatives (48.7 years vs. 41.7 years, P = 0.29); however, neither difference reached significance.

**Table 3:** Details of patients carrying mutations in two or more genes.

ID	Age dx (yrs)	Gene	Associated syndrome or diseases	Nucleotide change	Protein change	Exon	FH of cancer and age of onset (yrs)
7	30	*BRCA2*	HBOCS	c.5699C>G	p.S1900*	11	Paternal aunt (Br,57)
		*FANCD2*	FA	c.4234_4239delAGTGAG	p.S1412_E1413del	43	Paternal aunt (Lung,58)
							Maternal grandfather (Lung,73)
22	37	*BRCA1*	HBOCS	c.2110_2111delAA	p.N704Cfs*7	11	Mother (Br,60; Lym,61)
		*MSH3*	LS	c.162_179del18AGTGAG	p.A57_A62del	1	Maternal aunt (Br,49)
		*MUTYH*	FAP2	c.850-2A>G	p.?		Maternal aunt (Br,34)
							Maternal aunt (Br,50)
							Maternal grandfather (Br,68)
24	26, 36	*BRCA2*	HBOCS	c.7142delC	p.P2381Hfs*13	14	Father (rectum,64)
		*WRN*	WS	c.4245dupT	p.D1416*	35	Paternal aunt (rectum,47)
							Paternal grandmother (stomach,87)
29	39	*BRCA1*	HBOCS	c.5503C>T	p.R1835*	24	Sister (Br,36)
		*TNFRSF13B*	CVID2	c.102delC	p.E36Kfs*48	5	Mother (Bladder,64)
							Maternal aunt (Br,54)
75	51	*MSH3*	LS	c.2305delG	p.V769*	16	Sister (Br,53)
		*RGSL1*	Br	c.1357C>T	p.Q453*	6	Father (Liver,51)
							Paternal aunt (Br, 75)
							Paternal aunt (Nasopharynx,89)
							Paternal uncle (Nasopharynx,75)
							Cousin (Br,57)
77	34	*RAD50*	Br, ovary	c.1291_1295delGAGAT	p.E431Kfs*3	9	Sister (Br,32)
		*CDKN2A*	Familial melanoma	c.480G>A	p.W160*	4	Mother (Br,41)
86	52	*RAD51C*	FA	c.343dupG	p.V115Gfs*24	2	Sister (Br,57)
		*SLX4*	FA	c.3583_3585delATT	p.I1195del	12	Mother (Ovary,63)
							Maternal uncle (Liver,45)
							Father (Lym,79)
							Paternal aunt (Lung,65)
							Grandfather (Stomach,81)

Mutation nomenclature follows the recommended guidelines of the Human Genome Variation Society with the nucleotide numbering based on GenBank reference sequence indicated by its accession NCBI number. Details were listed in S2 Table. Abbreviations and definitions are as follows: FH, family history; HBOCS, hereditary breast and ovarian cancer syndrome; Br, breast; FA, Fanconi anemia; Lym, lymphoma; LS, Lynch syndrome; FAP2, familial adenomatous polyposis, 2; WS, werner syndrome; and CVID2, common variable immune deficiency,2.

### Functional Analysis of *MSH3* Germline Mutation and Gene-Gene Interaction

**Fig 4:**
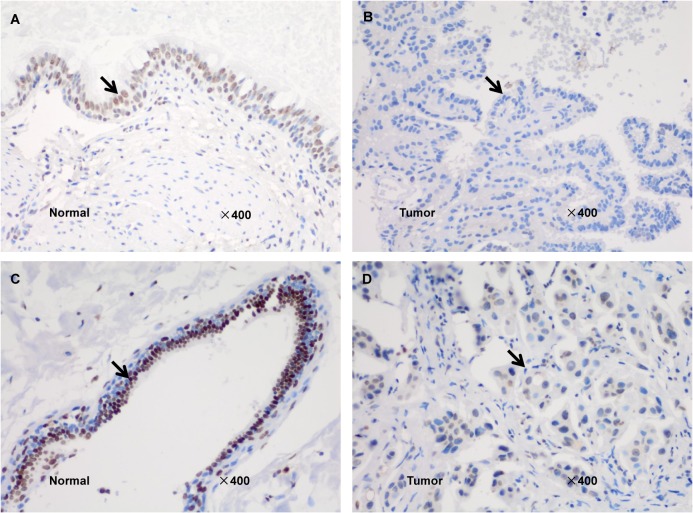
Expression of Msh3 protein in normal and tumor tissues of *MSH3* mutation carriers. Arrows indicate the location of the expression of Msh3 protein. A, the normal ovarian tissue of the proband from family No. 49. B, the ovarian tumor tissue of the proband from family No. 49. The loss of Msh3 expression was observed in the tumor tissue. C, the normal breast tissue of the sister of the proband from family No. 75. D, the breast tumor tissue of the sister of the proband from family No. 75. Msh3 is expressed strongly in the normal tissue and weakly in the tumor tissue.

In total, six paired (normal and tumor) breast tissue samples, one paired colon tissue sample and one paired ovarian tissue sample from three families with *MSH3* mutations were available for MSI and IHC analysis. An analysis of mononucleotide and dinucleotide repeat microsatellite markers identified two MSI-low (MSI-L) cases (25%) and six microsatellite stable (MSS) cases (75%). Seven of the eight cases exhibited mutations in at least one EMAST locus (Table 4). Msh3 expression was obviously lost in tumor tissue samples of two patients with MSH3 mutation (Fig 4 and Table 4) and studies have suggested that deficient *MMR* protein expression in tumor tissues were implications for the present of *MMR* gene mutations [30].

**Table 4:** Results of IHC and MSI analysis in tissues of *MSH3* mutation patients.

Family No.	Patient	Site of disease	MSH3 IHC Normal	MSH3 IHC Tumor	MSI BAT25	MSI BAT26	MSI D2S123	MSI D5S346	MSI D17S250	MSI D18S69	MSI D18S64	MSI MYCL1	MSI D20S82	MSI D9S242	MSI D20S85	MSI L17835	MSI D19S394	MSI D8S321	NCI	NCI and D status	EMAST status
22	Proband	Breast	3+	2+	**-**	**-**	**-**	**-**	**-**	**-**	**-**	**-**	**-**	**-**	**-**	**+**	**+**	**+**	S	S	E
22	Mother	Breast	2+	2+	**-**	**-**	**-**	**-**	**-**	**+**	**+**	**-**	**-**	**-**	**-**	**-**	**-**	**-**	S	L	non-E
49	Proband	Breast	3+	2+	**-**	**-**	**-**	**-**	**-**	**-**	**-**	**-**	**-**	**-**	**-**	**-**	**-**	**+**	S	S	E
49	Proband	Ovary	2+	0	**-**	**-**	**-**	**-**	**-**	**-**	**-**	**-**	**+**	**-**	**-**	**-**	**+**	**-**	S	S	E
49	Mother	Breast	1+	1+	**-**	**-**	**-**	**-**	**-**	**-**	**-**	**+**	**-**	**-**	**-**	**-**	**-**	**-**	S	S	E
49	Mother	Colon	2+	2+	**-**	**-**	**-**	**-**	**-**	**-**	**-**	**-**	**-**	**-**	**-**	**-**	**+**	**-**	S	S	E
75	Proband	Breast	3+	3+	**-**	**-**	**-**	**-**	**-**	**-**	**-**	**-**	**-**	**-**	**+**	**-**	**-**	**-**	S	S	E
75	Sister	Breast	3+	1+	**-**	**-**	**-**	**-**	**-**	**+**	**-**	**+**	**-**	**+**	**-**	**-**	**-**	**+**	S	L	E

For MSH3 IHC, “3+” indicates strong staining, “2+” indicates intermediate staining, “1+” indicates week staining, and “0” indicates no staining. For MSI profiles, it includes MSI data for five consensus National Cancer Institute (NCI) microsatellite markers (two mono-A repeats, BAT25 and BAT26, and three dinucleotide repeats, D2S123 through D17S250), two additional dinucleotide (D) markers (D18S69 and D18S64), and seven EMAST markers (MYCL1 through D8S321). For MSI data, “-” indicates the absence of the mutation, and “+” indicates the presence of the mutation. For MSI using the NCI panel (NCI), S indicates MSS. For MSI using the NCI panel and two additional dinucleotide markers (NCI and D status), S indicates MSS, and L indicates MSI-L. For EMAST status, E indicates EMAST positive, and non-E indicates EMAST negative.

**Fig 5:**
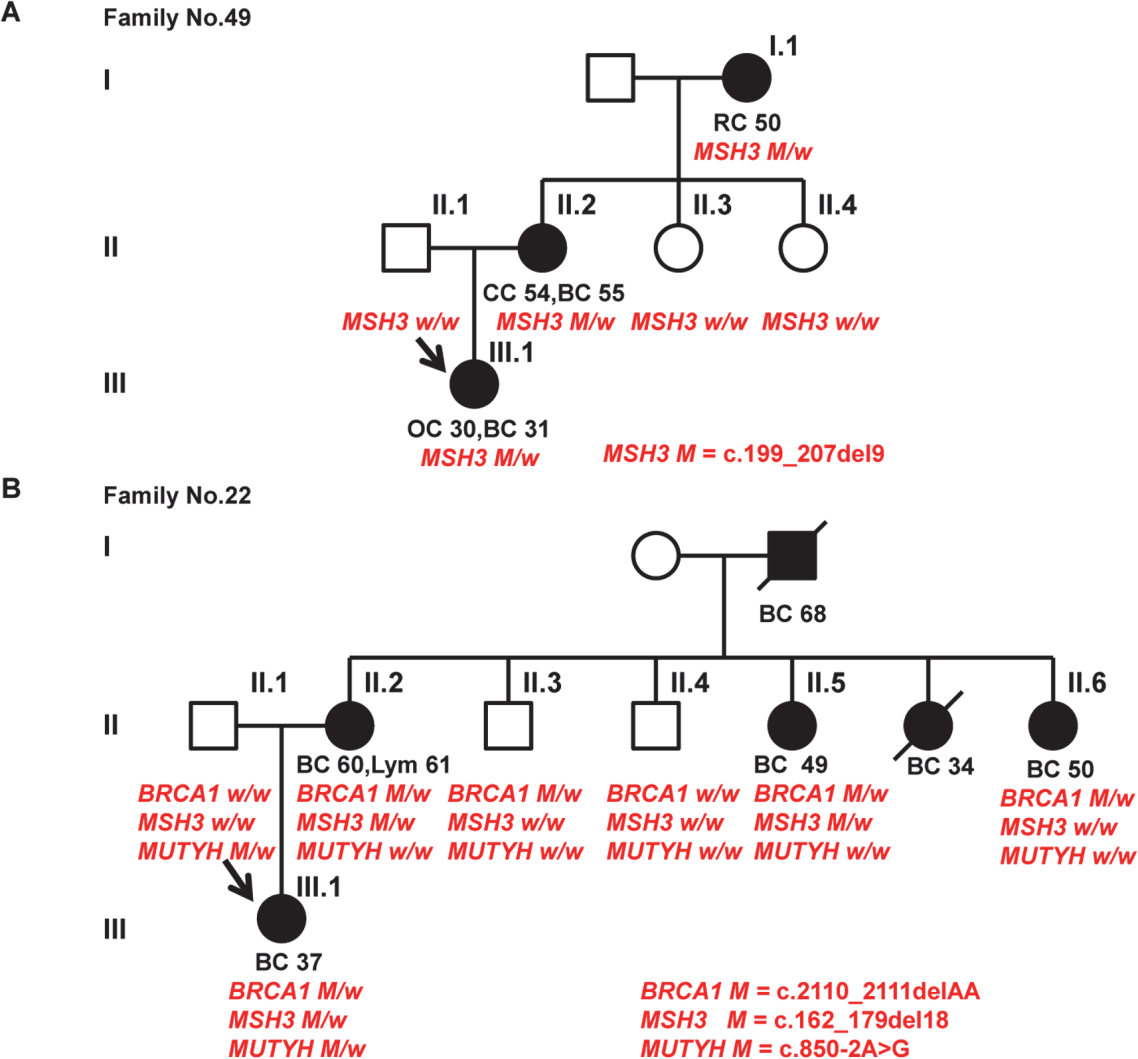
Pedigrees and results of segregation studies of family No. 49 and family No. 22. Subjects with cancers are indicated with solid symbols. Sites of cancer are breast (BC), ovary (OC), rectum (RC), colon (CC), and lymphoma (Lym). Ages under symbols indicate age at cancer diagnosis. Probands are indicated with arrows. Individuals having the gene mutation were indicated as *M/w*, and those not having such gene mutation were indicated as *w/w*. A, the pedigree and results of segregation analysis of family No. 49. B, the pedigree and results of segregation analysis of family No. 22.

Moreover, family No. 49 was identified as carrying a single *MSH3* germline mutation, and the family history manifested as typical LS. A segregation study indicated that this mutation segregated well with the diseases (Fig 5A). However, family No. 22 was identified as carrying both *MSH3* and *BRCA1* germline mutations, and the family history manifested as typical HBOCS instead of LS. A segregation study indicated that the *BRCA1* mutation completely segregated with the disease, whereas the *MSH3* mutation incompletely segregated with the disease (Fig 5B).

## Discussion

We performed comprehensive analysis of 152 genes associated with hereditary cancer in Chinese families who were at high risk for breast cancer, using targeted capture and NGS. We identified 34 (34/99, 34.3%) families who carried putative deleterious germline mutations. *BRCA2* mutations were more common than *BRCA1* mutations in our study, which is consistent with other Chinese studies [31, 32]. Two recurrent mutations were separately detected in two unrelated breast cancer patients in our study, and we have previously reported that the *BRCA1* c.5468-1_5474del8 mutation, together with another recurrent mutation, 1100delAT, accounted for 34.8% of all *BRCA1* mutations [33]. The founder effects of these recurrent mutations in *BRCA1* and *BRCA2* require further investigation.

Two topics regarding TP53 germline mutation detection are currently under debate. First, is the selection of patients for TP53 mutation analysis effective according to the LFS or the Chompret criteria [34]? According to our results, the *TP53* mutation detection rates in families fulfilling the classic LFS and the Chompret criteria were 50% and 9.1%, respectively. Previous studies have reported that the corresponding *TP53* mutation detection rates were approximately 56%-73% and 21%-36%, respectively [2, 35, 36]. More importantly, two of the three families with a *TP53* germline mutation met neither the classic LFS criteria nor the Chompret criteria. Other studies reported similar findings. Mouchawar et al. examined early-onset breast cancer patients and found that three of five *TP53* mutation carriers did not meet the classic LFS or LFL (Li-Fraumeni-like) syndrome criteria [7]. Second, is it necessary for early-onset (≤30 years) breast cancer patients to undergo *TP53* gene testing [37]? In a multiethnic Asian cohort, Lee et al. reported that the frequency of the *TP53* germline mutation was approximately 5% in breast cancer patients who were diagnosed at ≤35 years of age [38]. In an Australian population-based cohort, Mouchawar et al. found that 2/52 (4%) of very-early-onset (≤30 years) breast cancer patients who were unselected for family history carried a germline *TP53* mutation, and 3/42 (7%) of early-onset (31 to 39 years) breast cancer patients with a family history of breast or ovarian cancer carried a *TP53* germline mutation [7]. Our results indicated that 10% (2/20) of very-early-onset (≤30 years) breast cancer patients with a family history of cancer carried a *TP53* mutation, which is consistent with the results of Mouchawar et al. Since 2009, the NCCN guidelines have recommended that breast cancer patients who are diagnosed before the age of 30 and who are *BRCA1/2* negative should also be offered genetic counseling and testing for *TP53*, in addition to those fulfilling LFS or LFL syndrome criteria. Additionally, 2 of the 99 patients reported a family history of PTB and both carried a *TP53* germline mutation. Although PTB is not identified as a component of LFS according to the NCCN guidelines, Birch et al. [39] have reported that the detection rate of a *TP53* germline mutation in families with PTB was significantly high, which is consistent with our findings.

In the Chinese population, nearly all of the pathogenic mutations in LS families were identified in common *MMR* genes (*MSH2*, *MLH1* and *MSH6*) [40], which is different from that identified in our study. This difference may be attributed to the fact that, in current clinical practice, *MSH3* and *MLH3* are not routinely tested in patients suspected of having LS, but we conducted a comprehensive examination of multiple genes and obtained different results. The association between *MLH3* germline mutation and LS has been confirmed [41]. Meanwhile, it has been reported that *MSH3* deficiency is associated with EMAST and with low levels of instability at dinucleotide repeat loci [19], and *MSH3* deficiency may elevate susceptibility to certain neoplastic diseases and contribute to tumor initiation [42, 43]. In our study, an *MSH3* germline mutation was related to EMAST and MSI-L at dinucleotide repeat loci (Table 4), which is consistent with previous studies [19]. Moreover, we identified a typical LS family with an *MSH3* germline mutation, and segregation analysis indicated that this mutation segregated well with the familial diseases. Therefore, our findings suggested the association between *MSH3* germline mutation and LS. In our study, the mutation detection rate of common *MMR* genes in families fulfilling the revised Bethesda guidelines was 6.7% (*MLH1*: 1/15). In Western studies with large sample sizes, the corresponding common MMR gene mutation detection rate was only 2.5%-3.8% [44, 45]. Moreover, three (60%) of five *MMR* gene mutations were identified in patients who did not fulfill the revised Bethesda guidelines. Hampel and colleagues also found that approximately 28% of LS cases did not fulfill the revised Bethesda guidelines [8]. Although these guidelines, which were introduced in 2002, are more sensitive than the Amsterdam criteria in identifying individuals who are at risk for LS [46], the revised Bethesda guidelines still miss carriers.

A missense mutation c.1296 C>G (N432K) in CDH1 was detected in 1 of these 99 patients. This germline mutation has been previously reported in gastric cancer [47]. It can generate the E-cadherin exon9-skipping and was supposed to be a disease-causing mutation. The CDH1 gene appears to be rare in Chinese with breast cancer. Zhu et al. [48] found no germline disease associated mutations in a patient with familial diffuse gastric cancer and lobular breast cancer. More research is required in order to describe the role of CDH1 mutations in Chinese at high risk of breast cancer.

Except for BRCA1/2, FA pathway-related genes accounted for 28.6% (6/21) of the remaining genes containing loss-of-function mutations in our study. However, to date, three FA genes (FANCD1, FANCN, FANCJ) have been shown to be bona fide breast cancer susceptibility genes. The sequencing of DNA from members of families with heightened breast cancer risk has not revealed mutations in other FA genes [49]. It is unclear why some FA gene mutations impose a higher risk of cancer than others. We also detected heterozygous germline mutations in three genes that follow an autosomal recessive mode of pathogenesis, which include *WRN*, *MUTYH*, and *CYP17A1*. It is generally believed that recessive genes are not pathogenic in heterozygotes. However, some studies have reported that heterozygous mutations of certain genes are associated with increased cancer risk. Wang et al. [27] reported the association of a *WRN* heterozygous mutation with breast cancer susceptibility in Chinese women. Nielsen et al. observed a significantly increased risk of breast cancer in female *MUTYH* associated polyposis coli patients [28]. Hopper et al. [29] detected a deleterious *CYP17A1* germline mutation, p.R239X, in three sisters with early-onset breast cancer, and the breast cancer risk associated with this mutation has been reported to be more than 20 times the population incidence.

A high proportion (20.6%, 7/34) of patients carrying mutations in two or more genes was detected in our study. Walsh et al. stated that a single deleterious mutation in any one of the breast cancer susceptibility genes is sufficient to significantly increase breast cancer risk [5]. However, different findings have emerged as high-throughput sequencing technology has increased in popularity. Whole-exome sequencing studies conducted in *BRCA1/2*-negative familial breast cancer patients revealed that each sequenced individual harbored as many as 35 deleterious germline mutations on average [50]. Due to the difficulty inherent in functional studies, determining which mutations are pathogenic is problematic. In our study, all the tested genes are hereditary cancer susceptibility genes, therefore, their germline mutations can be considered more relevant to breast cancer risk. However, the analysis of gene-gene interactions is also complex. Currently, there are few systematic studies of gene-gene interactions among breast cancer susceptibility genes. Lavie et al. [51] reported that when compared to those carrying a single *BRCA1* or *BRCA2* mutation, women with double mutations had a much younger mean age at breast cancer diagnosis, which is similar to our findings. However, the effect of gene-gene interactions is not only confined to synergism. It is possible that antagonism may also exist, as indicated in this study (see Fig 5). Borg et al. [52] identified a family with both *MLH1* and *BRCA1* mutations, and they found that the double heterozygotes developed breast cancer but not colorectal or endometrial cancer. These authors proposed that the *BRCA1* deficiency had a potential protective effect against carcinogenic events in the colon. Thus, gene-gene interactions are very complex and require further study.

Pleiotropy occurs when one gene has an effect on multiple phenotypes. Sivakumaran et al. performed a systematic review and reported pleiotropy is a common property of genes and SNPs associated with disease traits [53]. Indeed, we observed several incidents where relatives from the same family developed different cancer types (see Fig 1), suggesting a potential role of pleiotropy. Even though it is hard to test for pleiotropy with our limited sample size, it is a crucial component of disease etiology which will need further investigation.

In summary, we identified comprehensive genetic factors for Chinese hereditary breast cancer using NGS. Our findings reconfirmed the importance of traditional genetic testing based on NCCN guidelines because almost all high-penetrance gene mutations were detected in the families that fulfill the typical phenotypes of hereditary cancer syndromes listed in the NCCN guidelines. Furthermore, NGS also identified a few gene mutations in the families not fulfill the NCCN guidelines, as indicated in our study (see Table 2), especially in families carrying mutations in two or more genes, in which a potential gene mutation does not present typical phenotypes (see Fig 5). So we think NGS may be a supplement of traditional genetic counseling. We also identified a lot of low-penetrance gene mutations which clinical significance has not been fully cleared yet, and these findings provide valuable epidemiological information for the future studies. Large sample sizes and multi-center population should be studied in the future to obtain more comprehensive data. We believe that our extensive findings will be helpful to the development of breast cancer genetic counseling in China.

## Supporting Information

S1 Table List of genes tested in this study. (DOCX)

S2 Table Genomic information of genes referenced in this study. (DOCX)

S3 Table Next generation sequencing metrics and coverage. (DOCX)
