# Supplementary material for: Identification of a Comprehensive Spectrum of Genetic Factors for Hereditary Breast Cancer in a Chinese Population by Next-Generation Sequencing
Source: PLoS One. 2015 Apr 30;10(4):e0125571. doi: 10.1371/journal.pone.0125571 (PMC4415911; doi:10.1371/journal.pone.0125571)
Supplement: S1 Table — (DOCX) [file pone.0125571.s001.docx]

**Table S1** List of genes tested in this study.

| **Gene** | **Syndrome** | **Reference** |
| --- | --- | --- |
| *ADA* | Adenosine Deaminase Deficiency | Arrendondo-Vega FX, Santisteban I, Notarangelo LD, El DJ, Buckley R, et al. (1998) Seven novel mutations in the adenosine deaminase (ADA) gene in patients with severe and delayed onset combined immunodeficiency: G74C, V129M, G140E, R149W, Q199P, 462delG, and E337del. Mutations in brief no. 142. Online. Hum Mutat 11: 482. doi: 10.1002/ (SICI) 1098-1004 (1998) 11: 6 < 482: :AID-HUMU15>3.0.CO;2-E |
| *ALK* | Neuroblastoma, Familial | Mosse YP, Laudenslager M, Longo L, Cole KA, Wood A, et al. (2008) Identification of ALK as a major familial neuroblastoma predisposition gene. Nature 455: 930-935.doi:10.1038/nature07261 |
| *APC* | Familial adenomatous polyposis | Powell SM, Petersen GM, Krush AJ, Booker S, Jen J, et al. (1993) Molecular diagnosis of familial adenomatous polyposis. N Engl J Med 329: 1982-1987. doi: 10.1056/NEJM199312303292702 |
| *ARL11* |  | Sellick GS, Catovsky D, Houlston RS (2006) Familial cancer associated with a polymorphism in ARLTS1. N Engl J Med 354: 1204-1205, 1204-1205. doi: 10.1056/NEJMc053522 |
| *ATM* | Ataxia Telangiectasia | Swift M, Morrell D, Massey RB, Chase CL (1991) Incidence of cancer in 161 families affected by ataxia-telangiectasia. N Engl J Med 325: 1831-1836. doi: 10.1056/NEJM199112263252602 |
| *ATR* | Oropharyngeal cancer syndrome | Tanaka A, Weinel S, Nagy N, O'Driscoll M, Lai-Cheong JE, et al. (2012) Germline mutation in ATR in autosomal- dominant oropharyngeal cancer syndrome. Am J Hum Genet 90: 511-517.doi:10.1016/j.ajhg.2012.01.007 |
| *BABAM1* |  | Zheng Y, Zhang J, Niu Q, Olopade OI, Huo D (2011) Germline mutational analysis of the C19orf62 gene in African-American women with breast cancer. Breast Cancer Res Treat 127:871-877.doi:10.1007/s10549-011-1445-y |
| *BARD1* |  | Karppinen SM, Heikkinen K, Rapakko K, Winqvist R (2004) Mutation screening of the BARD1 gene: evidence for involvement of the Cys557Ser allele in hereditary susceptibility to breast cancer. J Med Genet 41: e114.doi:10.1136/jmg.2004.020669 |
| *BLM* | Bloom syndrome | German J (1969) Bloom's syndrome. I. Genetical and clinical observations in the first twenty-seven patients. Am J Hum Genet 21: 196-227 |
| *BMPR1A* | Juvenile polyposis | Howe JR, Bair JL, Sayed MG, Anderson ME, Mitros FA, et al. (2001) Germline mutations of the gene encoding bone morphogenetic protein receptor 1A in juvenile polyposis. Nat Genet 28: 184-187.doi:10.1038/88919 |
| *BRCA1* | Breast-ovarian cancer syndrome | Miki Y, Swensen J, Shattuck-Eidens D, Futreal PA, Harshman K, et al. (1994) A strong candidate for the breast and ovarian cancer susceptibility gene BRCA1. Science 266: 66-71 |
| *BRCA2* | Breast-ovarian cancer syndrome / Fanconi anemia | Wooster R, Neuhausen SL, Mangion J, Quirk Y, Ford D, et al. (1994) Localization of a breast cancer susceptibility gene, BRCA2, to chromosome 13q12-13. Science 265: 2088-2090 |
| *BRIP1* | Fanconi anemia | Levitus M, Waisfisz Q, Godthelp BC, de Vries Y, Hussain S, et al. (2005) The DNA helicase BRIP1 is defective in Fanconi anemia complementation group J. Nat Genet 37: 934-935.doi:10.1038/ng1625 |
| *CASP10* | Autoimmune Lymphoproliferative Syndrome | Wang J, Zheng L, Lobito A, Chan FK, Dale J, et al. (1999) Inherited human Caspase 10 mutations underlie defective lymphocyte and dendritic cell apoptosis in autoimmune lymphoproliferative syndrome type II. Cell 98: 47-58.doi:10.1016/S0092-8674(00)80605-4 |
| *CASP8* | Autoimmune Lymphoproliferative Syndrome | Chun HJ, Zheng L, Ahmad M, Wang J, Speirs CK, et al. (2002) Pleiotropic defects in lymphocyte activation caused by caspase-8 mutations lead to human immunodeficiency. Nature 419: 395-399.doi:10.1038/nature01063 |
| *CDH1* | Hereditary diffuse gastric cancer | Guilford P, Hopkins J, Harraway J, McLeod M, McLeod N, et al. (1998) E-cadherin germline mutations in familial gastric cancer. Nature 392: 402-405.doi:10.1038/32918 |
| *CDK4* | Melanoma,Familial | Zuo L, Weger J, Yang Q, Goldstein AM, Tucker MA, et al. (1996) Germline mutations in the p16INK4a binding domain of CDK4 in familial melanoma. Nat Genet 12: 97-99.doi:10.1038/ng0196-97 |
| *CDKN1B* |  | Georgitsi M, Raitila A, Karhu A, van der Luijt RB, Aalfs CM, et al. (2007) Germline CDKN1B/p27Kip1 mutation in multiple endocrine neoplasia. J Clin Endocrinol Metab 92: 3321-3325.doi:10.1210/jc.2006-2843 |
| *CDKN1C* | Beckwith-wiedemann snydrome | Hatada I, Nabetani A, Morisaki H, Xin Z, Ohishi S, et al. (1997) New p57KIP2 mutations in Beckwith-Wiedemann syndrome. Hum Genet 100: 681-683 |
| *CDKN2A* | Melanoma,Familial | Kamb A, Shattuck-Eidens D, Eeles R, Liu Q, Gruis NA, et al. (1994) Analysis of the p16 gene (CDKN2) as a candidate for the chromosome 9p melanoma susceptibility locus. Nat Genet 8: 23-26.doi:10.1038/ng0994-22 |
| *CFTR* | Hereditary pancreatitis | Cohn JA, Friedman KJ, Noone PG, Knowles MR, Silverman LM, et al. (1998) Relation between mutations of the cystic fibrosis gene and idiopathic pancreatitis. N Engl J Med 339:653-658.doi:10.1056/NEJM199809033391002 |
| *CHEK2* |  | Walsh T, Casadei S, Coats KH, Swisher E, Stray SM, et al. (2006) Spectrum of mutations in BRCA1, BRCA2, CHEK2, and TP53 in families at high risk of breast cancer. JAMA 295: 1379-1388.doi:10.1001/jama.295.12.1379 |
| *c-KIT* | Familial gastrointestinal stromal tumor | Nishida T, Hirota S, Taniguchi M, Hashimoto K, Isozaki K, et al. (1998) Familial gastrointestinal stromal tumours with germline mutation of the KIT gene. Nat Genet 19: 323-324.doi:10.1038/1209 |
| *CLSPN* |  | Erkko H, Pylkas K, Karppinen SM, Winqvist R (2008) Germline alterations in the CLSPN gene in breast cancer families. Cancer Lett 261: 93-97.doi:10.1016/j.canlet.2007.11.003 |
| *CTCF* |  | Zhou XL, Werelius B, Lindblom A (2004) A screen for germline mutations in the gene encoding CCCTC-binding factor (CTCF) in familial non-BRCA1/BRCA2 breast cancer. Breast Cancer Res 6: R187-R190.doi:10.1186/bcr774 |
| *CYP11A1* |  | Setiawan VW, Cheng I, Stram DO, Giorgi E, Pike MC, et al. (2006) A systematic assessment of common genetic variation in CYP11A and risk of breast cancer. Cancer Res 66: 12019-12025.doi:10.1158/0008-5472.CAN-06-1101 |
| *CYP17A1* |  | Hopper JL, Hayes VM, Spurdle AB, Chenevix-Trench G, Jenkins MA, et al. (2005) A protein-truncating mutation in CYP17A1 in three sisters with early-onset breast cancer. Hum Mutat 26: 298-302.doi:10.1002/humu.20237 |
| *CYP19A1* |  | Haiman CA, Stram DO, Pike MC, Kolonel LN, Burtt NP, et al. (2003) A comprehensive haplotype analysis of CYP19 and breast cancer risk: the Multiethnic Cohort. Hum Mol Genet 12: 2679-2692.doi:10.1093/hmg/ddg294 |
| *CYP1B1* |  | Jeannot E, Poussin K, Chiche L, Bacq Y, Sturm N, et al. (2007) Association of CYP1B1 germ line mutations with hepatocyte nuclear factor 1alpha-mutated hepatocellular adenoma. Cancer Res 67: 2611-2616.doi:10.1158/0008-5472.CAN-06-3947 |
| *DAPK1* |  | Raval A, Tanner SM, Byrd JC, Angerman EB, Perko JD, et al. (2007) Downregulation of death-associated protein kinase 1 (DAPK1) in chronic lymphocytic leukemia. Cell 129: 879-890.doi:10.1016/j.cell.2007.03.043 |
| *DDB2* | Xeroderma pigmentosum | Oh KS, Khan SG, Jaspers NG, Raams A, Ueda T, et al. (2006) Phenotypic heterogeneity in the XPB DNA helicase gene (ERCC3): xeroderma pigmentosum without and with Cockayne syndrome. Hum Mutat 27: 1092-1103.doi:10.1002/humu.20392 |
| *ELAC2* | Hereditary prostate cancer | Tavtigian SV, Simard J, Teng DH, Abtin V, Baumgard M, et al. (2001) A candidate prostate cancer susceptibility gene at chromosome 17p. Nat Genet 27: 172-180.doi:10.1038/84808 |
| *ENG* | Juvenile polyposis | Sweet K, Willis J, Zhou XP, Gallione C, Sawada T, et al. (2005) Molecular classification of patients with unexplained hamartomatous and hyperplastic polyposis. JAMA 294: 2465-2473.doi:10.1001/jama.294.19.2465 |
| *EPCAM* | Hereditary nonpolyposis colon cancer syndrome | Kuiper RP, Vissers LE, Venkatachalam R, Bodmer D, Hoenselaar E, et al. (2011) Recurrence and variability of germline EPCAM deletions in Lynch syndrome. Hum Mutat 32: 407-414.doi:10.1002/humu.21446 |
| *EPHB2* | Hereditary prostate cancer | Huusko P, Ponciano-Jackson D, Wolf M, Kiefer JA, Azorsa DO, et al. (2004) Nonsense-mediated decay microarray analysis identifies mutations of EPHB2 in human prostate cancer. Nat Genet 36: 979-983.doi:10.1038/ng1408 |
| *ERBB2* |  | McKean-Cowdin R, Kolonel LN, Press MF, Pike MC, Henderson BE (2001) Germ-line HER-2 variant and breast cancer risk by stage of disease. Cancer Res 61: 8393-8394 |
| *ERCC2* | Xeroderma pigmentosum | Broughton BC, Berneburg M, Fawcett H, Taylor EM, Arlett CF, et al. (2001) Two individuals with features of both xeroderma pigmentosum and trichothiodystrophy highlight the complexity of the clinical outcomes of mutations in the XPD gene. Hum Mol Genet 10: 2539-2547 |
| *ERCC3* | Xeroderma pigmentosum | Oh KS, Khan SG, Jaspers NG, Raams A, Ueda T, et al. (2006) Phenotypic heterogeneity in the XPB DNA helicase gene (ERCC3): xeroderma pigmentosum without and with Cockayne syndrome. Hum Mutat 27: 1092-1103.doi:10.1002/humu.20392 |
| *ERCC4* | Xeroderma pigmentosum | Sijbers AM, van Voorst VP, Snoek JW, Raams A, Jaspers NG, et al. (1998) Homozygous R788W point mutation in the XPF gene of a patient with xeroderma pigmentosum and late-onset neurologic disease. J Invest Dermatol 110: 832-836.doi:10.1046/j.1523-1747.1998.00171.x |
| *ERCC5* | Xeroderma pigmentosum | Nouspikel T, Clarkson SG (1994) Mutations that disable the DNA repair gene XPG in a xeroderma pigmentosum group G patient. Hum Mol Genet 3: 963-967 |
| *ERCC6* | Cockayne's syndrome | Troelstra C, van Gool A, de Wit J, Vermeulen W, Bootsma D, et al. (1992) ERCC6, a member of a subfamily of putative helicases, is involved in Cockayne's syndrome and preferential repair of active genes. Cell 71: 939-953 |
| *EXT1* | Hereditary Multiple Osteochondromas | Philippe C, Porter DE, Emerton ME, Wells DE, Simpson AH, et al. (1997) Mutation screening of the EXT1 and EXT2 genes in patients with hereditary multiple exostoses. Am J Hum Genet 61: 520-528 |
| *EXT2* | Hereditary Multiple Osteochondromas | Philippe C, Porter DE, Emerton ME, Wells DE, Simpson AH, et al. (1997) Mutation screening of the EXT1 and EXT2 genes in patients with hereditary multiple exostoses. Am J Hum Genet 61: 520-528 |
| *FAM175A* |  | Osorio A, Barroso A, Garcia MJ, Martinez-Delgado B, Urioste M, et al. (2009) Evaluation of the BRCA1 interacting genes RAP80 and CCDC98 in familial breast cancer susceptibility. Breast Cancer Res Treat 113: 371-376.doi:10.1007/s10549-008-9933-4 |
| *FANCA* | Fanconi anemia | Fanconi anaemia/Breast cancer consortium (1996) Fanconi anaemia/Breast cancer consortium.Positional cloning of the Fanconi anaemia group A gene. Nat Genet 14(3):324-328. |
| *FANCB* | Fanconi anemia | Meetei AR, Levitus M, Xue Y, Medhurst AL, Zwaan M, et al. (2004) X-linked inheritance of Fanconi anemia complementation group B. Nat Genet 36: 1219-1224.doi:10.1038/ng1458 |
| *FANCC* | Fanconi anemia | Strathdee CA, Gavish H, Shannon WR, Buchwald M (1992) Cloning of cDNAs for Fanconi's anaemia by functional complementation. Nature 358: 434.doi:10.1038/358434a0 |
| *FANCD2* | Fanconi anemia | Timmers C, Taniguchi T, Hejna J, Reifsteck C, Lucas L, et al. (2001) Positional cloning of a novel Fanconi anemia gene, FANCD2. Mol Cell 7: 241-248 |
| *FANCE* | Fanconi anemia | de Winter JP, Rooimans MA, van Der Weel L, van Berkel CG, Alon N, et al. (2000) The Fanconi anaemia gene FANCF encodes a novel protein with homology to ROM. Nat Genet 24: 15-16.doi:10.1038/71626 |
| *FANCF* | Fanconi anemia | de Winter JP, Rooimans MA, van Der Weel L, van Berkel CG, Alon N, et al. (2000) The Fanconi anaemia gene FANCF encodes a novel protein with homology to ROM. Nat Genet 24: 15-16.doi:10.1038/71626 |
| *FANCG* | Fanconi anemia | de Winter JP, Rooimans MA, van Der Weel L, van Berkel CG, Alon N, et al. (2000) The Fanconi anaemia gene FANCF encodes a novel protein with homology to ROM. Nat Genet 24: 15-16.doi:10.1038/71626 |
| *FANCI* | Fanconi anemia | Dorsman JC, Levitus M, Rockx D, Rooimans MA, Oostra AB, et al. (2007) Identification of the Fanconi anemia complementation group I gene, FANCI. Cell Oncol 29: 211-218 |
| *FANCL* | Fanconi anemia | Meetei AR, de Winter JP, Medhurst AL, Wallisch M, Waisfisz Q, et al. (2003) A novel ubiquitin ligase is deficient in Fanconi anemia. Nat Genet 35: 165-170.doi:10.1038/ng1241 |
| *FANCM* | Fanconi anemia | Meetei AR, Medhurst AL, Ling C, Xue Y, Singh TR, et al. (2005) A human ortholog of archaeal DNA repair protein Hef is defective in Fanconi anemia complementation group M. Nat Genet 37: 958-963.doi:10.1038/ng1626 |
| *FAS* | Autoimmune Lymphoproliferative Syndrome | Rieux-Laucat F, Le Deist F, Hivroz C, Roberts IA, Debatin KM, et al. (1995) Mutations in Fas associated with human lymphoproliferative syndrome and autoimmunity. Science 268: 1347-1349 |
| *FASL* | Autoimmune Lymphoproliferative Syndrome | Del-Rey M, Ruiz-Contreras J, Bosque A, Calleja S, Gomez-Rial J, et al. (2006) A homozygous Fas ligand gene mutation in a patient causes a new type of autoimmune lymphoproliferative syndrome. Blood 108: 1306-1312.doi:10.1182/blood-2006-04-015776 |
| *FGFR3* | Muenke syndrome | Bellus GA, Gaudenz K, Zackai EH, Clarke LA, Szabo J, et al. (1996) Identical mutations in three different fibroblast growth factor receptor genes in autosomal dominant craniosynostosis syndromes. Nat Genet 14: 174-176.doi:10.1038/ng1096-174 |
| *FH* | Hereditary leiomyomatosis renal cell cancer syndrome | Toro JR, Nickerson ML, Wei MH, Warren MB, Glenn GM, et al. (2003) Mutations in the fumarate hydratase gene cause hereditary leiomyomatosis and renal cell cancer in families in North America. Am J Hum Genet 73: 95-106.doi:10.1086/376435 |
| *FHIT* |  | Ahmadian M, Wistuba II, Fong KM, Behrens C, Kodagoda DR, et al. (1997) Analysis of the FHIT gene and FRA3B region in sporadic breast cancer, preneoplastic lesions, and familial breast cancer probands. Cancer Res 57: 3664-3668 |
| *FLCN* | Birt-hogg-dube syndrome | Schmidt LS, Nickerson ML, Warren MB, Glenn GM, Toro JR, et al. (2005) Germline BHD-mutation spectrum and phenotype analysis of a large cohort of families with Birt-Hogg-Dube syndrome. Am J Hum Genet 76: 1023-1033.doi:10.1086/430842 |
| *GATA3* |  | Usary J, Llaca V, Karaca G, Presswala S, Karaca M, et al. (2004) Mutation of GATA3 in human breast tumors. Oncogene 23: 7669-7678.doi:10.1038/sj.onc.1207966 |
| *GPC3* | Simpson-Golabi-Behmel syndrome | Pilia G, Hughes-Benzie RM, MacKenzie A, Baybayan P, Chen EY, et al. (1996) Mutations in GPC3, a glypican gene, cause the Simpson-Golabi-Behmel overgrowth syndrome. Nat Genet 12: 241-247.doi:10.1038/ng0396-241 |
| *H19* | Beckwith-wiedemann snydrome /Wilms tumor,Familial/Silver-Russell syndrome | Sparago A, Cerrato F, Vernucci M, Ferrero GB, Silengo MC, et al. (2004) Microdeletions in the human H19 DMR result in loss of IGF2 imprinting and Beckwith-Wiedemann syndrome. Nat Genet 36: 958-960.doi:10.1038/ng1410 |
| *HFE* |  | Syrjakoski K, Fredriksson H, Ikonen T, Kuukasjarvi T, Autio V, et al. (2006) Hemochromatosis gene mutations among Finnish male breast and prostate cancer patients. Int J Cancer 118: 518-520.doi:10.1002/ijc.21331 |
| *IGF2* | Beckwith-wiedemann snydrome | Ohlsson R, Nystrom A, Pfeifer-Ohlsson S, Tohonen V, Hedborg F, et al. (1993) IGF2 is parentally imprinted during human embryogenesis and in the Beckwith-Wiedemann syndrome. Nat Genet 4: 94-97.doi:10.1038/ng0593-94 |
| *IL2RG* | Severe combined immune deficiency | Conley ME, Lavoie A, Briggs C, Brown P, Guerra C, et al. (1988) Nonrandom X chromosome inactivation in B cells from carriers of X chromosome-linked severe combined immunodeficiency. Proc Natl Acad Sci U S A 85: 3090-3094 |
| *IL7R* | Severe combined immune deficiency | Puel A, Ziegler SF, Buckley RH, Leonard WJ (1998) Defective IL7R expression in T(-)B(+)NK(+) severe combined immunodeficiency. Nat Genet 20: 394-397.doi:10.1038/3877 |
| *KCNQ1* | Beckwith-wiedemann snydrome | Lee MP, Hu RJ, Johnson LA, Feinberg AP (1997) Human KVLQT1 gene shows tissue-specific imprinting and encompasses Beckwith-Wiedemann syndrome chromosomal rearrangements. Nat Genet 15: 181-185.doi:10.1038/ng0297-181 |
| *KCNQ1OT1* | Beckwith-wiedemann snydrome | Weksberg R, Nishikawa J, Caluseriu O, Fei YL, Shuman C, et al. (2001) Tumor development in the Beckwith-Wiedemann syndrome is associated with a variety of constitutional molecular 11p15 alterations including imprinting defects of KCNQ1OT1. Hum Mol Genet 10: 2989-3000 |
| *KRAS* |  | Paranjape T, Heneghan H, Lindner R, Keane FK, Hoffman A, et al. (2011) A 3'-untranslated region KRAS variant and triple-negative breast cancer: a case-control and genetic analysis. Lancet Oncol 12: 377-386.doi:10.1016/S1470-2045(11)70044-4 |
| *MC1R* |  | Bastiaens MT, ter Huurne JA, Kielich C, Gruis NA, Westendorp RG, et al. (2001) Melanocortin-1 receptor gene variants determine the risk of nonmelanoma skin cancer independently of fair skin and red hair. Am J Hum Genet 68: 884-894 |
| *MEN1* | Multiple endocrine neoplasia, Type 1 | Larsson C, Skogseid B, Oberg K, Nakamura Y, Nordenskjold M (1988) Multiple endocrine neoplasia type 1 gene maps to chromosome 11 and is lost in insulinoma. Nature 332: 85-87.doi:10.1038/332085a0 |
| *MET* | Renal cancer syndrome,Hereditary Papillary | Schmidt L, Duh FM, Chen F, Kishida T, Glenn G, et al. (1997) Germline and somatic mutations in the tyrosine kinase domain of the MET proto-oncogene in papillary renal carcinomas. Nat Genet 16: 68-73.doi:10.1038/ng0597-68 |
| *MLH1* | Hereditary non-polyposis colon cancer syndrome/Familial adenomatous polyposis | Bronner CE, Baker SM, Morrison PT, Warren G, Smith LG, et al. (1994) Mutation in the DNA mismatch repair gene homologue hMLH1 is associated with hereditary non-polyposis colon cancer. Nature 368: 258-261.doi:10.1038/368258a0 |
| *MLH3* | Hereditary non-polyposis colon cancer | Wu Y, Berends MJ, Sijmons RH, Mensink RG, Verlind E, et al. (2001) A role for MLH3 in hereditary nonpolyposis colorectal cancer. Nat Genet 29: 137-138.doi:10.1038/ng1001-137 |
| *MRE11A* |  | Walsh T, Casadei S, Lee MK, Pennil CC, Nord AS, et al. (2011) Mutations in 12 genes for inherited ovarian, fallopian tube, and peritoneal carcinoma identified by massively parallel sequencing. Proc Natl Acad Sci U S A 108: 18032-18037.doi:10.1073/pnas.1115052108 |
| *MSH2* | Hereditary non-polyposis colon cancer syndrome | Wijnen J, van der Klift H, Vasen H, Khan PM, Menko F, et al. (1998) MSH2 genomic deletions are a frequent cause of HNPCC. Nat Genet 20: 326-328.doi:10.1038/3795 |
| *MSH3* | Hereditary non-polyposis colon cancer syndrome | Liu B, Parsons R, Papadopoulos N, Nicolaides NC, Lynch HT, et al. (1996) Analysis of mismatch repair genes in hereditary non-polyposis colorectal cancer patients. Nat Med 2: 169-174 |
| *MSH6* | Hereditary non-polyposis colon cancer syndrome | Miyaki M, Konishi M, Tanaka K, Kikuchi-Yanoshita R, Muraoka M, et al. (1997) Germline mutation of MSH6 as the cause of hereditary nonpolyposis colorectal cancer. Nat Genet 17: 271-272.doi:10.1038/ng1197-271 |
| *MSX1* |  | Sliwinski T, Synowiec E, Czarny P, Gomulak P, Forma E, et al. (2010) The c.469+46_56del mutation in the homeobox MSX1 gene--a novel risk factor in breast cancer? Cancer Epidemiol 34: 652-655.doi:10.1016/j.canep.2010.06.003 |
| *MUTYH* | MYH-associated polyposis/Familial adenomatous polyposis | Sieber OM, Lipton L, Crabtree M, Heinimann K, Fidalgo P, et al. (2003) Multiple colorectal adenomas, classic adenomatous polyposis, and germ-line mutations in MYH. N Engl J Med 348: 791-799.doi:10.1056/NEJMoa025283 |
| *MYC* |  | Callahan R, Campbell G (1989) Mutations in human breast cancer: an overview. J Natl Cancer Inst 81: 1780-1786 |
| *NBN* | Nijmegen breakage syndrome | Varon R, Vissinga C, Platzer M, Cerosaletti KM, Chrzanowska KH, et al. (1998) Nibrin, a novel DNA double-strand break repair protein, is mutated in Nijmegen breakage syndrome. Cell 93: 467-476 |
| *NF1* | Neurofibromatosis, Type 1 | Wallace MR, Marchuk DA, Andersen LB, Letcher R, Odeh HM, et al. (1990) Type 1 neurofibromatosis gene: identification of a large transcript disrupted in three NF1 patients. Science 249: 181-186 |
| *NF2* | Neurofibromatosis, Type 2 | Trofatter JA, MacCollin MM, Rutter JL, Murrell JR, Duyao MP, et al. (1993) A novel moesin-, ezrin-, radixin-like gene is a candidate for the neurofibromatosis 2 tumor suppressor. Cell 75: 826 |
| *NOD2* |  | Huzarski T, Lener M, Domagala W, Gronwald J, Byrski T, et al. (2005) The 3020insC allele of NOD2 predisposes to early-onset breast cancer. Breast Cancer Res Treat 89: 91-93.doi:10.1007/s10549-004-1250-y |
| *PALB2* | Fanconi anemia | Reid S, Schindler D, Hanenberg H, Barker K, Hanks S, et al. (2007) Biallelic mutations in PALB2 cause Fanconi anemia subtype FA-N and predispose to childhood cancer. Nat Genet 39: 162-164.doi:10.1038/ng1947 |
| *PALLD* | Hereditary pancreatic cancer susceptibility | Pogue-Geile KL, Chen R, Bronner MP, Crnogorac-Jurcevic T, Moyes KW, et al. (2006) Palladin mutation causes familial pancreatic cancer and suggests a new cancer mechanism. PLoS Med 3: e516.doi:10.1371/journal.pmed.0030516 |
| *PARP1* |  | Durocher F, Labrie Y, Ouellette G, Simard J (2007) Genetic sequence variations and ADPRT haplotype analysis in French Canadian families with high risk of breast cancer. J Hum Genet 52: 963-977.doi:10.1007/s10038-007-0203-9 |
| *PDGFRA* | Familial gastrointestinal stromal tumor | Chompret A, Kannengiesser C, Barrois M, Terrier P, Dahan P, et al. (2004) PDGFRA germline mutation in a family with multiple cases of gastrointestinal stromal tumor. Gastroenterology 126: 318-321 |
| *PHOX2B* | Neuroblastoma, Familial | Bourdeaut F, Trochet D, Janoueix-Lerosey I, Ribeiro A, Deville A, et al. (2005) Germline mutations of the paired-like homeobox 2B (PHOX2B) gene in neuroblastoma. Cancer Lett 228: 51-58.doi:10.1016/j.canlet.2005.01.055 |
| *PIK3CA* | Megalencephaly-Capillary Malformation Syndrome | Riviere JB, Mirzaa GM, O'Roak BJ, Beddaoui M, Alcantara D, et al. (2012) De novo germline and postzygotic mutations in AKT3, PIK3R2 and PIK3CA cause a spectrum of related megalencephaly syndromes. Nat Genet 44: 934-940.doi:10.1038/ng.2331 |
| *PMS1* | Hereditary nonpolyposis colon cancer syndrome | Nicolaides NC, Papadopoulos N, Liu B, Wei YF, Carter KC, et al. (1994) Mutations of two PMS homologues in hereditary nonpolyposis colon cancer. Nature 371: 75-80.doi:10.1038/371075a0 |
| *PMS2* | Hereditary nonpolyposis colon cancer syndrome/Familial adenomatous polyposis | Nicolaides NC, Papadopoulos N, Liu B, Wei YF, Carter KC, et al. (1994) Mutations of two PMS homologues in hereditary nonpolyposis colon cancer. Nature 371: 75-80.doi:10.1038/371075a0 |
| *POLH* | Xeroderma pigmentosum | Ben RM, Messaoud O, Mebazaa A, Riahi O, Azaiez H, et al. (2011) A novel POLH gene mutation in a xeroderma pigmentosum-V Tunisian patient: phenotype-genotype correlation. J Genet 90: 483-487 |
| *POU6F2* | Wilms tumor,Familial | Perotti D, De Vecchi G, Testi MA, Lualdi E, Modena P, et al. (2004) Germline mutations of the POU6F2 gene in Wilms tumors with loss of heterozygosity on chromosome 7p14. Hum Mutat 24: 400-407.doi:10.1002/humu.20096 |
| *PRKAR1A* | Carney complex | Stratakis CA, Kirschner LS, Taymans SE, Carney JA, Basson CT (1999) Genetic heterogeneity in Carney complex (OMIM 160980): contributions of loci at chromosomes 2 and 17 in its genetics. Am J Hum Genet 65:A447. |
| *PTCH1* | Nevoid basal cell carcinoma syndrome | Smyth I, Narang MA, Evans T, Heimann C, Nakamura Y, et al. (1999) Isolation and characterization of human patched 2 (PTCH2), a putative tumour suppressor gene inbasal cell carcinoma and medulloblastoma on chromosome 1p32. Hum Mol Genet 8: 291-297 |
| *PTEN* | PTEN hamartoma syndrome(PHS) | Zhou X, Hampel H, Thiele H, Gorlin RJ, Hennekam RC, et al. (2001) Association of germline mutation in the PTEN tumour suppressor gene and Proteus and Proteus-like syndromes. Lancet 358: 210-211 |
| *PTPRC* | Severe combined immune deficiency | Porcu M, Kleppe M, Gianfelici V, Geerdens E, De Keersmaecker K, et al. (2012) Mutation of the receptor tyrosine phosphatase PTPRC (CD45) in T-cell acute lymphoblastic leukemia. Blood 119: 4476-4479.doi:10.1182/blood-2011-09-379958 |
| *RAD50* |  | Heikkinen K, Karppinen SM, Soini Y, Makinen M, Winqvist R (2003) Mutation screening of Mre11 complex genes: indication of RAD50 involvement in breast and ovarian cancer susceptibility. J Med Genet 40: e131 |
| *RAD51* |  | Kato M, Yano K, Matsuo F, Saito H, Katagiri T, et al. (2000) Identification of Rad51 alteration in patients with bilateral breast cancer. J Hum Genet 45: 133-137.doi:10.1007/s100380050199 |
| *RAD51C* | Fanconi anemia | Vaz F, Hanenberg H, Schuster B, Barker K, Wiek C, et al. (2010) Mutation of the RAD51C gene in a Fanconi anemia-like disorder. Nat Genet 42: 406-409.doi:10.1038/ng.570 |
| *RAD51D* |  | Loveday C, Turnbull C, Ramsay E, Hughes D, Ruark E, et al. (2011) Germline mutations in RAD51D confer susceptibility to ovarian cancer. Nat Genet 43: 879-882.doi:10.1038/ng.893 |
| *RAG1* | Severe combined immune deficiency | Corneo B, Moshous D, Gungor T, Wulffraat N, Philippet P, et al. (2001) Identical mutations in RAG1 or RAG2 genes leading to defective V(D)J recombinase activity can cause either T-B-severe combined immune deficiency or Omenn syndrome. Blood 97: 2772-2776 |
| *RAG2* | Severe combined immune deficiency | Corneo B, Moshous D, Gungor T, Wulffraat N, Philippet P, et al. (2001) Identical mutations in RAG1 or RAG2 genes leading to defective V(D)J recombinase activity can cause either T-B-severe combined immune deficiency or Omenn syndrome. Blood 97: 2772-2776 |
| *RB1* | Retinoblastoma, Hereditary | Lohmann DR, Brandt B, Hopping W, Passarge E, Horsthemke B (1996) The spectrum of RB1 germ-line mutations in hereditary retinoblastoma. Am J Hum Genet 58: 940-949 |
| *RB1CC1* |  | Chano T, Kontani K, Teramoto K, Okabe H, Ikegawa S (2002) Truncating mutations of RB1CC1 in human breast cancer. Nat Genet 31: 285-288.doi:10.1038/ng911 |
| *RECQL4* | Rothmund-thomson syndrome/Baller-Gerold Syndrome/RAPADILINO syndrome | Kitao S, Shimamoto A, Goto M, Miller RW, Smithson WA, et al. (1999) Mutations in RECQL4 cause a subset of cases of Rothmund-Thomson syndrome. Nat Genet 22: 82-84.doi:10.1038/8788 |
| *RET* | Mutiple endocrine neoplasia,Type 2 | Mulligan LM, Kwok JB, Healey CS, Elsdon MJ, Eng C, et al. (1993) Germ-line mutations of the RET proto-oncogene in multiple endocrine neoplasia type 2A. Nature 363: 458-460.doi:10.1038/363458a0 |
| *RGS16* |  | Wiechec E, Wiuf C, Overgaard J, Hansen LL (2011) High-resolution melting analysis for mutation screening of RGSL1, RGS16, and RGS8 in breast cancer. Cancer Epidemiol Biomarkers Prev 20: 397-407.doi:10.1158/1055-9965.EPI-10-0514 |
| *RGS8* |  | Wiechec E, Wiuf C, Overgaard J, Hansen LL (2011) High-resolution melting analysis for mutation screening of RGSL1, RGS16, and RGS8 in breast cancer. Cancer Epidemiol Biomarkers Prev 20: 397-407.doi:10.1158/1055-9965.EPI-10-0514 |
| *RGSL1* |  | Wiechec E, Wiuf C, Overgaard J, Hansen LL (2011) High-resolution melting analysis for mutation screening of RGSL1, RGS16, and RGS8 in breast cancer. Cancer Epidemiol Biomarkers Prev 20: 397-407.doi:10.1158/1055-9965.EPI-10-0514 |
| *RHOBTB2* |  | Hamaguchi M, Meth JL, von Klitzing C, Wei W, Esposito D, et al. (2002) DBC2, a candidate for a tumor suppressor gene involved in breast cancer. Proc Natl Acad Sci U S A 99: 13647-13652.doi:10.1073/pnas.212516099 |
| *RNASEL* | Hereditary prostate cancer | Rokman A, Ikonen T, Seppala EH, Nupponen N, Autio V, et al. (2002) Germline alterations of the RNASEL gene, a candidate HPC1 gene at 1q25, in patients and families with prostate cancer. Am J Hum Genet 70: 1299-1304.doi:10.1086/340450 |
| *RPL11* | Diamond-Blackfan Anemia | Gazda HT, Sheen MR, Vlachos A, Choesmel V, O'Donohue MF, et al. (2008) Ribosomal protein L5 and L11 mutations are associated with cleft palate and abnormal thumbs in Diamond-Blackfan anemia patients. Am J Hum Genet 83: 769-780.doi:10.1016/j.ajhg.2008.11.004 |
| *RPL35A* | Diamond-Blackfan Anemia | Farrar JE, Nater M, Caywood E, McDevitt MA, Kowalski J, et al. (2008) Abnormalities of the large ribosomal subunit protein, Rpl35a, in Diamond-Blackfan anemia. Blood 112: 1582-1592.doi:10.1182/blood-2008-02-140012 |
| *RPL5* | Diamond-Blackfan Anemia | Gazda HT, Sheen MR, Vlachos A, Choesmel V, O'Donohue MF, et al. (2008) Ribosomal protein L5 and L11 mutations are associated with cleft palate and abnormal thumbs in Diamond-Blackfan anemia patients. Am J Hum Genet 83: 769-780.doi:10.1016/j.ajhg.2008.11.004 |
| *RPS10* | Diamond-Blackfan Anemia | Doherty L, Sheen MR, Vlachos A, Choesmel V, O'Donohue MF, et al. (2010) Ribosomal protein genes RPS10 and RPS26 are commonly mutated in Diamond-Blackfan anemia. Am J Hum Genet 86: 222-228.doi:10.1016/j.ajhg.2009.12.015 |
| *RPS17* | Diamond-Blackfan Anemia | Cmejla R, Cmejlova J, Handrkova H, Petrak J, Pospisilova D (2007) Ribosomal protein S17 gene (RPS17) is mutated in Diamond-Blackfan anemia. Hum Mutat 28: 1178-1182.doi:10.1002/humu.20608 |
| *RPS19* | Diamond-Blackfan Anemia | Draptchinskaia N, Gustavsson P, Andersson B, Pettersson M, Willig TN, et al. (1999) The gene encoding ribosomal protein S19 is mutated in Diamond-Blackfan anaemia. Nat Genet 21: 169-175.doi:10.1038/5951 |
| *RPS24* | Diamond-Blackfan Anemia | Gazda HT, Grabowska A, Merida-Long LB, Latawiec E, Schneider HE, et al. (2006) Ribosomal protein S24 gene is mutated in Diamond-Blackfan anemia. Am J Hum Genet 79: 1110-1118.doi:10.1086/510020 |
| *RPS26* | Diamond-Blackfan Anemia | Doherty L, Sheen MR, Vlachos A, Choesmel V, O'Donohue MF, et al. (2010) Ribosomal protein genes RPS10 and RPS26 are commonly mutated in Diamond-Blackfan anemia. Am J Hum Genet 86: 222-228.doi:10.1016/j.ajhg.2009.12.015 |
| *RPS7* | Diamond-Blackfan Anemia | Gazda HT, Sheen MR, Vlachos A, Choesmel V, O'Donohue MF, et al. (2008) Ribosomal protein L5 and L11 mutations are associated with cleft palate and abnormal thumbs in Diamond-Blackfan anemia patients. Am J Hum Genet 83: 769-780.doi:10.1016/j.ajhg.2008.11.004 |
| *SDHA* | Paraganglioma-Pheochromocytoma syndrome,hereditary | Burnichon N, Briere JJ, Libe R, Vescovo L, Riviere J, et al. (2010) SDHA is a tumor suppressor gene causing paraganglioma. Hum Mol Genet 19: 3011-3020.doi:10.1093/hmg/ddq206 |
| *SDHAF2* | Paraganglioma-Pheochromocytoma syndrome,hereditary | Hao HX, Khalimonchuk O, Schraders M, Dephoure N, Bayley JP, et al. (2009) SDH5, a gene required for flavination of succinate dehydrogenase, is mutated in paraganglioma. Science 325: 1139-1142.doi:10.1126/science.1175689 |
| *SDHB* | Familial gastrointestinal stromal tumor/Paraganglioma-Pheochromocytoma syndrome, hereditary | Astuti D, Latif F, Dallol A, Dahia PL, Douglas F, et al. (2001) Gene mutations in the succinate dehydrogenase subunit SDHB cause susceptibility to familial pheochromocytoma and to familial paraganglioma. Am J Hum Genet 69: 49-54.doi:10.1086/321282 |
| *SDHC* | Familial gastrointestinal stromal tumor/Paraganglioma-Pheochromocytoma syndrome,hereditary | Niemann S, Muller U (2000) Mutations in SDHC cause autosomal dominant paraganglioma, type 3. Nat Genet 26: 268-270.doi:10.1038/81551 |
| *SDHD* | Paraganglioma-Pheochromocytoma syndrome,hereditary | Baysal BE, Ferrell RE, Willett-Brozick JE, Lawrence EC, Myssiorek D, et al. (2000) Mutations in SDHD, a mitochondrial complex II gene, in hereditary paraganglioma. Science 287: 848-851 |
| *SH2D1A* | X-linked Lymphoproliferative syndrome | Arico M, Imashuku S, Clementi R, Hibi S, Teramura T, et al. (2001) Hemophagocytic lymphohistiocytosis due to germline mutations in SH2D1A, the X-linked lymphoproliferative disease gene. Blood 97: 1131-1133 |
| *SLX4* | Fanconi anemia | Kim Y, Lach FP, Desetty R, Hanenberg H, Auerbach AD, et al. (2011) Mutations of the SLX4 gene in Fanconi anemia. Nat Genet 43: 142-146.doi:10.1038/ng.750 |
| *SMAD4* | Juvenile polyposis | Howe JR, Roth S, Ringold JC, Summers RW, Jarvinen HJ, et al. (1998) Mutations in the SMAD4/DPC4 gene in juvenile polyposis. Science 280: 1086-1088 |
| *SMARCB1* | Rhabdoid predisposition syndrome | Versteege I, Sevenet N, Lange J, Rousseau-Merck MF, Ambros P, et al. (1998) Truncating mutations of hSNF5/INI1 in aggressive paediatric cancer. Nature 394: 203-206.doi:10.1038/28212 |
| *SMYD3* |  | Tsuge M, Hamamoto R, Silva FP, Ohnishi Y, Chayama K, et al. (2005) A variable number of tandem repeats polymorphism in an E2F-1 binding element in the 5' flanking region of SMYD3 is a risk factor for human cancers. Nat Genet 37: 1104-1107.doi:10.1038/ng1638 |
| *SPINK1* | Hereditary pancreatitis | Le Marechal C, Chen JM, Le Gall C, Plessis G, Chipponi J, et al. (2004) Two novel severe mutations in the pancreatic secretory trypsin inhibitor gene (SPINK1) cause familial and/or hereditary pancreatitis. Hum Mutat 23: 205.doi:10.1002/humu.9212 |
| *STK11* | Peutz-Jeghers syndrome | Hemminki A, Markie D, Tomlinson I, Avizienyte E, Roth S, et al. (1998) A serine/threonine kinase gene defective in Peutz-Jeghers syndrome. Nature 391: 184-187.doi:10.1038/34432 |
| *SULT1E1* |  | Cohen S, Laitman Y, Kaufman B, Milgrom R, Nir U, et al. (2009) SULT1E1 and ID2 genes as candidates for inherited predisposition to breast and ovarian cancer in Jewish women. Fam Cancer 8: 135-144.doi:10.1007/s10689-008-9218-4 |
| *TGFBR1* |  | Chen T, Jackson CR, Link A, Markey MP, Colligan BM, et al. (2006) Int7G24A variant of transforming growth factor-beta receptor type I is associated with invasive breast cancer. Clin Cancer Res 12: 392-397.doi:10.1158/1078-0432.CCR-05-1518 |
| *TMEM127* | Paraganglioma-Pheochromocytoma syndrome,hereditary | Yao L, Schiavi F, Cascon A, Qin Y, Inglada-Perez L, et al. (2010) Spectrum and prevalence of FP/TMEM127 gene mutations in pheochromocytomas and paragangliomas. JAMA 304: 2611-2619.doi:10.1001/jama.2010.1830 |
| *TNFRSF13B* | Common variable immune deficiency | Salzer U, Chapel HM, Webster AD, Pan-Hammarstrom Q, Schmitt-Graeff A, et al. (2005) Mutations in TNFRSF13B encoding TACI are associated with common variable immunodeficiency in humans. Nat Genet 37: 820-828.doi:10.1038/ng1600 |
| *TOPBP1* |  | Karppinen SM, Erkko H, Reini K, Pospiech H, Heikkinen K, et al. (2006) Identification of a common polymorphism in the TopBP1 gene associated with hereditary susceptibility to breast and ovarian cancer. Eur J Cancer 42: 2647-2652.doi:10.1016/j.ejca.2006.05.030 |
| *TP53* | Li-fraumeni syndrome | Malkin D (2011) Li-fraumeni syndrome. Genes Cancer 2: 475-484.doi:10.1177/1947601911413466 |
| *TSC1* | Tuberous sclerosis complex | van Slegtenhorst M, de Hoogt R, Hermans C, Nellist M, Janssen B, et al. (1997) Identification of the tuberous sclerosis gene TSC1 on chromosome 9q34. Science 277: 805-808 |
| *TSC2* | Tuberous sclerosis complex | European Chromosome 16 Tuberous Sclerosis Consortium (1993) Identification and characterization of the tuberous sclerosis gene on chromosome 16. Cell 75:1305-1315 |
| *TWIST1* | Saethre-Chotzen syndrome | Paznekas WA, Cunningham ML, Howard TD, Korf BR, Lipson MH, et al. (1998) Genetic heterogeneity of Saethre-Chotzen syndrome, due to TWIST and FGFR mutations. Am J Hum Genet 62: 1370-1380.doi:10.1086/301855 |
| *UIMC1* |  | Akbari MR, Ghadirian P, Robidoux A, Foumani M, Sun Y, et al. (2009) Germline RAP80 mutations and susceptibility to breast cancer. Breast Cancer Res Treat 113: 377-381.doi:10.1007/s10549-008-9938-z |
| *VHL* | Von hippel lindau syndrome | Seizinger BR, Rouleau GA, Ozelius LJ, Lane AH, Farmer GE, et al. (1988) Von Hippel-Lindau disease maps to the region of chromosome 3 associated with renal cell carcinoma. Nature 332: 268-269.doi:10.1038/332268a0 |
| *WAS* | Wiskott-Aldrich syndrome, X-linked thrombocytopenia, X-linked congenital neutropenia | Derry JM, Ochs HD, Francke U (1994) Isolation of a novel gene mutated in Wiskott-Aldrich syndrome. Cell 79: 922 |
| *WRN* | Werner syndrome | Yu CE, Oshima J, Fu YH, Wijsman EM, Hisama F, et al. (1996) Positional cloning of the Werner's syndrome gene. Science 272: 258-262 |
| *WT1* | Wilms tumor,Familial | Pelletier J, Bruening W, Li FP, Haber DA, Glaser T, et al. (1991) WT1 mutations contribute to abnormal genital system development and hereditary Wilms' tumour. Nature 353: 431-434.doi:10.1038/353431a0 |
| *XPA* | Xeroderma pigmentosum | Hirai Y, Kodama Y, Moriwaki S, Noda A, Cullings HM, et al. (2006) Heterozygous individuals bearing a founder mutation in the XPA DNA repair gene comprise nearly 1% of the Japanese population. Mutat Res 601: 171-178.doi:10.1016/j.mrfmmm.2006.06.010 |
| *XPC* | Xeroderma pigmentosum | Cartault F, Nava C, Malbrunot AC, Munier P, Hebert JC, et al. (2011) A new XPC gene splicing mutation has lead to the highest worldwide prevalence of xeroderma pigmentosum in black Mahori patients. DNA Repair (Amst) 10: 577-585.doi:10.1016/j.dnarep.2011.03.005 |
