# Supplementary material for: Identification of a Comprehensive Spectrum of Genetic Factors for Hereditary Breast Cancer in a Chinese Population by Next-Generation Sequencing
Source: PLoS One. 2015 Apr 30;10(4):e0125571. doi: 10.1371/journal.pone.0125571 (PMC4415911; doi:10.1371/journal.pone.0125571)
Supplement: S2 Table — (DOCX) [file pone.0125571.s002.docx]

**Table S2** Genomic information of genes referenced in this study.

| Gene | Genomic DNA | mRNA | Protein |
| --- | --- | --- | --- |
| *BRCA1* | NC_000017.11 | NM_007294.3 | NP_009225.1 |
| *BRCA2* | NC_000013.1 | NM_000059.3 | NP_000050.2 |
| *TP53* | NC_000017.1 | NM_000546.5 | NP_000537.3 |
| *MLH1* | NC_000003.11 | NM_000249.3 | NP_000240.1 |
| *MLH3* | NC_000014.8 | NM_001040108.1 | NP_001035197.1 |
| *MSH3* | NC_000005.9 | NM_002439.4 | NP_002430.3 |
| *CDH1* | NC_000016.9 | NM_004360.3 | NP_004351.1 |
| *RAD51C* | NC_000017.1 | NM_002876.3 | NP_002867.1 |
| *RAD50* | NC_000005.9 | NM_005732.3 | NP_005723.2 |
| *PALB2* | NC_000016.9 | NM_024675.3 | NP_078951.2 |
| *FANCD2* | NC_000003.11 | NM_001018115.1 | NP_001018125.1 |
| *FANCI* | NC_000015.9 | NM_018193.2 | NP_060663.2 |
| *SLX4* | NC_000016.9 | NM_032444.2 | NP_115 820.2 |
| *RGSL1* | NC_000001.1 | NM_001137669.1 | NP_001131141.1 |
| *CDKN2A* | NC_000009.11 | NM_001195132.1 | NP_001182061.1 |
| *SPINK1* | NC_000005.9 | NM_003122.3 | NP_003113.2 |
| *TNFRSF13B* | NC_000017.10 | NM_012452.2 | NP_036584.1 |
| *FGFR3* | NC_000004.11 | NM_022965.3 | NP_075254.1 |
| *WRN* | NC_000008.10 | NM_000553.4 | NP_000544.2 |
| *MUTYH* | NC_000001.1 | NM_001048174.1 | NP_001041639.1 |
| *CYP17A1* | NC_000010.1 | NM_000102.3 | NP_000093.1 |
