# Supplementary material for: Identification of a Comprehensive Spectrum of Genetic Factors for Hereditary Breast Cancer in a Chinese Population by Next-Generation Sequencing
Source: PLoS One. 2015 Apr 30;10(4):e0125571. doi: 10.1371/journal.pone.0125571 (PMC4415911; doi:10.1371/journal.pone.0125571)
Supplement: S3 Table — (DOCX) [file pone.0125571.s003.docx]

| **Table S3** Next generation sequencing metrics and coverage. | | | | | | | | | | | | |
| --- | --- | --- | --- | --- | --- | --- | --- | --- | --- | --- | --- | --- |
| Sample ID | Total effective reads | No. of reads uniquely mapped to genome | No. of reads uniquely mapped to target | Fraction of uniquely mapped on target | Effective sequences on target(Mb) | Fraction of effective bases on target | Average sequencing depth on target | Base covered on target | Coverage of target region | Base covered near target | Coverage of near target region | Fraction of target covered with at least 20x |
| 1 | 2589606 | 2432651 | 876313 | 36.00% | 64.71 | 28.30% | 104.1 | 613347 | 98.70% | 792282 | 97.60% | 92.10% |
| 2 | 2037957 | 1933083 | 902687 | 46.70% | 67.59 | 37.30% | 108.73 | 611540 | 98.40% | 785950 | 96.80% | 90.70% |
| 3 | 2123974 | 2006104 | 863125 | 43.00% | 64.54 | 34.30% | 103.82 | 609436 | 98.00% | 775012 | 95.50% | 89.50% |
| 4 | 1961516 | 1859631 | 867566 | 46.70% | 65.09 | 37.50% | 104.71 | 609762 | 98.10% | 775371 | 95.50% | 89.80% |
| 5 | 2353758 | 2219530 | 937603 | 42.20% | 70.44 | 33.60% | 113.32 | 609953 | 98.10% | 777216 | 95.80% | 89.60% |
| 6 | 2247902 | 2117515 | 916501 | 43.30% | 68.74 | 34.40% | 110.59 | 610513 | 98.20% | 782431 | 96.40% | 90.70% |
| 7 | 2335198 | 2212456 | 893133 | 40.40% | 66.59 | 32.30% | 107.13 | 610054 | 98.10% | 782092 | 96.40% | 89.70% |
| 8 | 2413391 | 2274678 | 942407 | 41.40% | 70.45 | 33.00% | 113.34 | 611364 | 98.40% | 784218 | 96.60% | 90.50% |
| 9 | 2264154 | 2141728 | 921569 | 43.00% | 68.73 | 34.10% | 110.57 | 610607 | 98.20% | 777934 | 95.90% | 90.60% |
| 10 | 2237581 | 2116371 | 877309 | 41.50% | 65.73 | 33.10% | 105.75 | 609621 | 98.10% | 774031 | 95.40% | 89.10% |
| 11 | 2216424 | 2097123 | 962608 | 45.90% | 72.16 | 36.60% | 116.09 | 611343 | 98.30% | 786270 | 96.90% | 90.50% |
| 12 | 2961668 | 2795183 | 866253 | 31.00% | 64.04 | 24.40% | 103.02 | 613436 | 98.70% | 789917 | 97.30% | 91.80% |
| 13 | 2403227 | 2269204 | 887589 | 39.10% | 66.24 | 31.10% | 106.57 | 610321 | 98.20% | 781862 | 96.30% | 89.90% |
| 14 | 2507657 | 2360649 | 912538 | 38.70% | 68.17 | 30.70% | 109.67 | 609393 | 98.00% | 779573 | 96.10% | 89.40% |
| 15 | 2766171 | 2604572 | 881393 | 33.80% | 65.64 | 26.80% | 105.59 | 610243 | 98.20% | 779818 | 96.10% | 89.60% |
| 16 | 2660214 | 2505434 | 904991 | 36.10% | 68.06 | 28.80% | 109.49 | 609814 | 98.10% | 782951 | 96.50% | 89.70% |
| 17 | 2447712 | 2314307 | 968479 | 41.80% | 72.55 | 33.30% | 116.71 | 611150 | 98.30% | 784072 | 96.60% | 90.40% |
| 18 | 3048106 | 2857206 | 874129 | 30.60% | 65.43 | 24.10% | 105.26 | 611570 | 98.40% | 785090 | 96.70% | 89.90% |
| 19 | 1988213 | 1884933 | 899523 | 47.70% | 67.42 | 38.10% | 108.46 | 611200 | 98.30% | 781803 | 96.30% | 90.00% |
| 20 | 2467743 | 2328819 | 970694 | 41.70% | 72.81 | 33.10% | 117.13 | 610722 | 98.20% | 782169 | 96.40% | 89.80% |
| 21 | 3321491 | 3117598 | 881347 | 28.30% | 66.08 | 22.40% | 106.31 | 611022 | 98.30% | 780944 | 96.20% | 90.50% |
| 22 | 2296897 | 2179582 | 874684 | 40.10% | 65.28 | 32.10% | 105.01 | 609228 | 98.00% | 775492 | 95.60% | 89.20% |
| 23 | 2676804 | 2520565 | 888506 | 35.30% | 66.13 | 27.90% | 106.39 | 609961 | 98.10% | 780748 | 96.20% | 89.60% |
| 24 | 1836369 | 1735082 | 822931 | 47.40% | 61.54 | 37.80% | 99.01 | 609040 | 98.00% | 779716 | 96.10% | 88.80% |
| 25 | 2075069 | 1962092 | 864845 | 44.10% | 64.46 | 34.90% | 103.69 | 610584 | 98.20% | 784663 | 96.70% | 90.80% |
| 26 | 2216237 | 2089901 | 862556 | 41.30% | 64.48 | 32.90% | 103.73 | 609539 | 98.10% | 777915 | 95.90% | 89.00% |
| 27 | 2657849 | 2510670 | 970736 | 38.70% | 72.72 | 30.80% | 116.99 | 610337 | 98.20% | 783166 | 96.50% | 91.10% |
| 28 | 2205932 | 2081003 | 936923 | 45.00% | 70.14 | 35.90% | 112.84 | 609477 | 98.00% | 778165 | 95.90% | 90.00% |
| 29 | 2070070 | 1959901 | 875713 | 44.70% | 65.57 | 35.80% | 105.48 | 609712 | 98.10% | 773732 | 95.30% | 89.40% |
| 30 | 2227699 | 2093825 | 871174 | 41.60% | 65.12 | 33.00% | 104.76 | 610130 | 98.20% | 783281 | 96.50% | 89.40% |
| 31 | 2441064 | 2309491 | 982078 | 42.50% | 73.46 | 34.00% | 118.17 | 610903 | 98.30% | 783094 | 96.50% | 90.10% |
| 32 | 3354539 | 3144138 | 895318 | 28.50% | 67.1 | 22.50% | 107.95 | 610395 | 98.20% | 783123 | 96.50% | 90.50% |
| 33 | 2109974 | 1992946 | 862720 | 43.30% | 64.49 | 34.60% | 103.74 | 608957 | 98.00% | 778203 | 95.90% | 89.40% |
| 34 | 2049027 | 1933934 | 832212 | 43.00% | 62.39 | 34.40% | 100.36 | 609179 | 98.00% | 774886 | 95.50% | 88.70% |
| 35 | 2128365 | 2007562 | 867694 | 43.20% | 64.99 | 34.40% | 104.55 | 609395 | 98.00% | 777856 | 95.80% | 89.20% |
| 36 | 2688470 | 2525062 | 975576 | 38.60% | 73.03 | 30.60% | 117.48 | 611724 | 98.40% | 786603 | 96.90% | 90.90% |
| 37 | 2073961 | 1970631 | 887931 | 45.10% | 66.16 | 35.90% | 106.43 | 611773 | 98.40% | 790156 | 97.40% | 91.60% |
| 38 | 2242354 | 2115358 | 888452 | 42.00% | 66.48 | 33.50% | 106.96 | 610273 | 98.20% | 778049 | 95.90% | 89.50% |
| 39 | 2405116 | 2271032 | 898695 | 39.60% | 67.16 | 31.60% | 108.05 | 610654 | 98.20% | 780438 | 96.20% | 89.60% |
| 40 | 2422757 | 2276988 | 893367 | 39.20% | 66.77 | 31.20% | 107.41 | 610830 | 98.30% | 781281 | 96.30% | 89.80% |
| 41 | 2572278 | 2434866 | 898924 | 36.90% | 67.02 | 29.30% | 107.81 | 612113 | 98.50% | 790248 | 97.40% | 91.50% |
| 42 | 2939315 | 2775576 | 858640 | 30.90% | 63.42 | 24.40% | 102.02 | 612488 | 98.50% | 786187 | 96.90% | 91.20% |
| 43 | 3127943 | 2957269 | 874935 | 29.60% | 64.61 | 23.30% | 103.94 | 613175 | 98.60% | 792699 | 97.70% | 92.60% |
| 44 | 2384699 | 2244160 | 932724 | 41.60% | 70.16 | 33.00% | 112.87 | 610149 | 98.20% | 778871 | 96.00% | 89.20% |
| 45 | 2413602 | 2273004 | 907838 | 39.90% | 67.97 | 31.80% | 109.35 | 610039 | 98.10% | 778494 | 95.90% | 89.70% |
| 46 | 2679875 | 2514753 | 927306 | 36.90% | 69.15 | 29.20% | 111.24 | 610120 | 98.20% | 782753 | 96.40% | 89.80% |
| 47 | 2188585 | 2058258 | 885125 | 43.00% | 65.99 | 33.90% | 106.16 | 609689 | 98.10% | 781279 | 96.30% | 90.20% |
| 48 | 2616622 | 2463619 | 910735 | 37.00% | 68.29 | 29.40% | 109.85 | 610075 | 98.10% | 783739 | 96.60% | 90.30% |
| 49 | 2380990 | 2250255 | 880514 | 39.10% | 65.75 | 31.20% | 105.77 | 610762 | 98.30% | 782196 | 96.40% | 89.60% |
| 50 | 2196808 | 2074556 | 959794 | 46.30% | 71.94 | 36.80% | 115.74 | 611214 | 98.30% | 785965 | 96.80% | 90.80% |
| 51 | 2357372 | 2237857 | 937806 | 41.90% | 70.14 | 33.60% | 112.84 | 610608 | 98.20% | 782302 | 96.40% | 90.10% |
| 52 | 2589514 | 2434217 | 865633 | 35.60% | 64.1 | 28.00% | 103.12 | 611950 | 98.40% | 786984 | 97.00% | 91.40% |
| 53 | 2147861 | 2023237 | 877534 | 43.40% | 65.6 | 34.50% | 105.53 | 610264 | 98.20% | 781726 | 96.30% | 89.60% |
| 54 | 2654586 | 2499520 | 889796 | 35.60% | 66.28 | 28.20% | 106.63 | 603840 | 97.10% | 771223 | 95.00% | 87.70% |
| 55 | 2143845 | 2031671 | 957268 | 47.10% | 71.81 | 37.70% | 115.52 | 611479 | 98.40% | 786668 | 96.90% | 90.90% |
| 56 | 2361536 | 2217177 | 920821 | 41.50% | 68.85 | 33.00% | 110.77 | 611316 | 98.30% | 783076 | 96.50% | 89.90% |
| 57 | 2365179 | 2243006 | 935675 | 41.70% | 69.96 | 33.40% | 112.55 | 610211 | 98.20% | 779719 | 96.10% | 89.70% |
| 58 | 2411986 | 2278916 | 861402 | 37.80% | 64.36 | 30.10% | 103.53 | 610478 | 98.20% | 776968 | 95.70% | 89.30% |
| 59 | 2360449 | 2235737 | 901422 | 40.30% | 67.08 | 32.20% | 107.92 | 610822 | 98.30% | 784220 | 96.60% | 90.10% |
| 60 | 2231345 | 2103369 | 941826 | 44.80% | 70.59 | 35.70% | 113.57 | 610593 | 98.20% | 784717 | 96.70% | 90.50% |
| 61 | 2228406 | 2097989 | 944725 | 45.00% | 70.83 | 35.80% | 113.95 | 609543 | 98.10% | 778423 | 95.90% | 90.00% |
| 62 | 3035185 | 2859845 | 884417 | 30.90% | 66.16 | 24.50% | 106.43 | 610208 | 98.20% | 780685 | 96.20% | 90.10% |
| 63 | 3257400 | 3083397 | 870540 | 28.20% | 64.31 | 22.30% | 103.46 | 613688 | 98.70% | 793518 | 97.80% | 92.20% |
| 64 | 2547610 | 2410116 | 1020972 | 42.40% | 76.49 | 33.70% | 123.05 | 609602 | 98.10% | 781469 | 96.30% | 90.90% |
| 65 | 2330038 | 2190102 | 911202 | 41.60% | 68.28 | 33.00% | 109.85 | 612034 | 98.50% | 781876 | 96.30% | 90.80% |
| 66 | 2575919 | 2444214 | 889777 | 36.40% | 66.25 | 29.00% | 106.58 | 604506 | 97.20% | 771352 | 95.00% | 87.70% |
| 67 | 1935860 | 1818917 | 867926 | 47.70% | 64.93 | 37.90% | 104.46 | 610488 | 98.20% | 778734 | 96.00% | 89.30% |
| 68 | 3057371 | 2867796 | 881016 | 30.70% | 65.94 | 24.30% | 106.09 | 610696 | 98.20% | 784592 | 96.70% | 90.20% |
| 69 | 3082498 | 2885578 | 878283 | 30.40% | 65.76 | 24.00% | 105.8 | 611126 | 98.30% | 785188 | 96.70% | 90.00% |
| 70 | 2684723 | 2536140 | 890928 | 35.10% | 66.42 | 27.80% | 106.85 | 611047 | 98.30% | 787260 | 97.00% | 91.00% |
| 71 | 2446739 | 2308955 | 883645 | 38.30% | 65.88 | 30.40% | 105.98 | 603054 | 97.00% | 771665 | 95.10% | 87.60% |
| 72 | 2413814 | 2285032 | 926097 | 40.50% | 69.26 | 32.30% | 111.41 | 610806 | 98.30% | 782857 | 96.50% | 90.60% |
| 73 | 2214617 | 2093170 | 862427 | 41.20% | 64.48 | 32.90% | 103.74 | 610127 | 98.20% | 780678 | 96.20% | 89.30% |
| 74 | 2775194 | 2605275 | 875997 | 33.60% | 65.46 | 26.70% | 105.32 | 609299 | 98.00% | 779397 | 96.00% | 89.60% |
| 75 | 2327159 | 2195392 | 918023 | 41.80% | 68.92 | 33.30% | 110.87 | 610982 | 98.30% | 785022 | 96.70% | 90.60% |
| 76 | 2261156 | 2134818 | 876942 | 41.10% | 65.47 | 32.70% | 105.32 | 610769 | 98.30% | 783334 | 96.50% | 89.70% |
| 77 | 2432227 | 2282961 | 932082 | 40.80% | 69.68 | 32.40% | 112.09 | 610114 | 98.20% | 784154 | 96.60% | 90.30% |
| 78 | 2145404 | 2024817 | 930351 | 45.90% | 69.62 | 36.70% | 112 | 610278 | 98.20% | 780120 | 96.10% | 90.50% |
| 79 | 2108298 | 1993134 | 879323 | 44.10% | 65.94 | 35.30% | 106.09 | 609827 | 98.10% | 773727 | 95.30% | 88.90% |
| 80 | 2165341 | 2042884 | 917428 | 44.90% | 68.88 | 35.80% | 110.82 | 611971 | 98.40% | 785399 | 96.80% | 91.20% |
| 81 | 2488107 | 2352310 | 942644 | 40.10% | 70.54 | 32.00% | 113.48 | 610567 | 98.20% | 778082 | 95.90% | 89.50% |
| 82 | 2110313 | 2000887 | 889271 | 44.40% | 66.27 | 35.50% | 106.62 | 604020 | 97.20% | 767592 | 94.60% | 87.70% |
| 83 | 2252664 | 2127588 | 956171 | 44.90% | 71.93 | 35.90% | 115.71 | 609771 | 98.10% | 774865 | 95.50% | 89.50% |
| 84 | 2060481 | 1942873 | 894996 | 46.10% | 66.94 | 36.70% | 107.68 | 610139 | 98.20% | 778463 | 95.90% | 89.60% |
| 85 | 2221403 | 2094378 | 947024 | 45.20% | 70.94 | 36.10% | 114.12 | 610264 | 98.20% | 779498 | 96.00% | 90.10% |
| 86 | 2502295 | 2345923 | 877875 | 37.40% | 65.21 | 29.50% | 104.91 | 611132 | 98.30% | 786178 | 96.90% | 90.40% |
| 87 | 2417661 | 2274027 | 879849 | 38.70% | 65.69 | 30.70% | 105.68 | 610469 | 98.20% | 784069 | 96.60% | 90.10% |
| 88 | 2870755 | 2697976 | 890315 | 33.00% | 66.66 | 26.10% | 107.24 | 610936 | 98.30% | 784064 | 96.60% | 90.20% |
| 89 | 2269654 | 2138804 | 866474 | 40.50% | 64.77 | 32.20% | 104.2 | 610481 | 98.20% | 781227 | 96.30% | 89.50% |
| 90 | 2070314 | 1958128 | 887852 | 45.30% | 66.67 | 36.30% | 107.26 | 609018 | 98.00% | 768866 | 94.70% | 88.90% |
| 91 | 2011495 | 1899921 | 867197 | 45.60% | 64.87 | 36.50% | 104.36 | 609330 | 98.00% | 780301 | 96.10% | 89.50% |
| 92 | 3287675 | 3097326 | 889100 | 28.70% | 66.57 | 22.80% | 107.1 | 610165 | 98.20% | 780353 | 96.20% | 90.10% |
| 93 | 2167310 | 2050009 | 938105 | 45.80% | 69.84 | 36.20% | 112.35 | 610142 | 98.20% | 780189 | 96.10% | 91.00% |
| 94 | 2073991 | 1959730 | 879480 | 44.90% | 65.56 | 35.60% | 105.47 | 611178 | 98.30% | 787508 | 97.00% | 91.20% |
| 95 | 2007889 | 1903122 | 869998 | 45.70% | 64.83 | 36.30% | 104.3 | 610699 | 98.20% | 785102 | 96.70% | 91.20% |
| 96 | 2139156 | 2020312 | 833762 | 41.30% | 62.34 | 32.70% | 100.29 | 609462 | 98.00% | 774526 | 95.40% | 89.00% |
| 97 | 2012247 | 1907914 | 870650 | 45.60% | 65.12 | 36.30% | 104.76 | 609588 | 98.10% | 773150 | 95.30% | 89.50% |
| 98 | 1910811 | 1803636 | 770694 | 42.70% | 57.62 | 33.80% | 92.69 | 608434 | 97.90% | 770583 | 94.90% | 88.80% |
| 99 | 1978980 | 1868785 | 860140 | 46.00% | 64.28 | 36.50% | 103.41 | 608916 | 98.00% | 776024 | 95.60% | 89.10% |
